# Blocking IL-6 trans-signaling mitigates benzalkonium chloride-induced inflammation, oxidative imbalance, ER stress, and barrier disruption in human corneal epithelial cells

**DOI:** 10.1186/s40662-026-00513-0

**Published:** 2026-10-20

**Authors:** Karina Lax, Joshua Glass, Antonella Aliste, Ashok Sharma, Shruti Sharma

**Affiliations:** 1 Center for Biotechnology and Genomic Medicine, Medical College of Georgia, Augusta University, 1120 15th Street, Augusta, GA 30912 USA; 2 Department of Ophthalmology, Augusta University, Augusta, GA USA

**Keywords:** IL-6 trans-signaling, Benzalkonium chloride, Corneal epithelium, sgp130Fc, Dry eye disease

## Abstract

**Background:**

Benzalkonium chloride (BAC), a commonly used preservative in ophthalmic formulations, elicits a pro-inflammatory cytokine response and cytotoxic effects in corneal epithelial cells, contributing to ocular surface injuries associated with dry eye disease (DED). Interleukin-6 (IL-6) trans-signaling is a well-established pro-inflammatory pathway that contributes to endothelial and epithelial barrier dysfunction. Thus, this study investigated whether targeted inhibition of IL-6 trans-signaling with sgp130Fc could attenuate BAC-induced cytotoxic injury in corneal epithelial cells.

**Methods:**

Human telomerase-immortalized corneal epithelial (hTCEpi) cells were exposed to 0.001% BAC for 30 min in the presence or absence of 10 μg/mL sgp130Fc. Markers of inflammation, oxidative imbalance, endoplasmic reticulum (ER) stress, and epithelial barrier disruption were assessed using enzyme-linked immunosorbent assay, reverse transcriptase polymerase chain reaction, and western blot analyses. Reactive oxygen species (ROS), antioxidant capacity, and intracellular calcium flux were quantified using colorimetric, fluorescence-based, and luminescence-based assays. Epithelial barrier function was assessed using electric cell-substrate impedance sensing. Statistical analyses were performed using one-way analysis of variance followed by Tukey’s or Holm-Šídák multiple-comparison tests, with *P* < 0.05 considered statistically significant.

**Results:**

BAC exposure activated IL-6 trans-signaling as indicated by an increased sIL-6R/sgp130 ratio, reduced mIL-6R levels, and unchanged gp130 levels. BAC also increased secreted IL6 levels, *IL6RA* expression, and downstream inflammatory mediators *MCP1* and *ICAM1*, all of which were attenuated by sgp130Fc. BAC-induced oxidative imbalance was characterized by increased ROS and hydrogen peroxide production, reduced antioxidant enzymes, and diminished total antioxidant capacity, whereas sgp130Fc mitigated these effects. In addition, BAC increased intracellular calcium levels and ER stress markers, including *HSP72*, BiP, PERK, and Ero1α, and impaired epithelial barrier function as observed by reduced trans-epithelial electrical resistance and decreased β-catenin, occludin, and claudin-1 levels. sgp130Fc mitigated increases in intracellular calcium, partially alleviated ER stress responses, and preserved epithelial barrier integrity and junctional protein levels.

**Conclusions:**

These findings indicate that IL-6 trans-signaling contributes to BAC-induced corneal epithelial injury, suggesting that targeting this pathway may have therapeutic potential for DED and related ocular surface inflammatory disorders.

**Supplementary Information:**

The online version contains supplementary material available at https://doi.org/10.1186/s40662-026-00513-0.

## Background

Dry eye disease (DED) is a multifactorial disorder characterized by tear film instability and loss of ocular surface homeostasis [1]. A central feature of DED is disruption of the corneal epithelial barrier which normally protects the ocular surface, maintains tear film stability, and regulates inflammatory signaling [2]. This barrier is maintained by intercellular junctional complexes including tight junctions and adherens junctions [3–5]. During ocular surface stress, chronic inflammation disrupts barrier-associated proteins, and both the production of excessive reactive oxygen species (ROS) and impaired antioxidant defenses further compromise epithelial integrity [2]. Together, these processes promote a self-sustaining cycle of ocular surface inflammation, oxidative damage, and epithelial dysfunction [2, 6, 7].

Benzalkonium chloride (BAC) is widely used as a preservative in ophthalmic formulations because of its broad-spectrum antimicrobial activity and surfactant properties [8, 9]. However, prolonged BAC exposure is associated with ocular surface toxicity and can induce pathological changes, including epithelial damage, inflammatory activation, oxidative injury, and impaired barrier function, thereby contributing to the development or exacerbation of DED [10–12]. Clinical studies have shown that discontinuation of BAC-preserved medications significantly improves dry eye symptoms and ocular surface staining [13]. As a cationic surfactant, BAC destabilizes cellular membranes, increases membrane permeability, and weakens corneal epithelial barrier integrity [14–16]. Although these effects are well documented, the signaling pathways that connect BAC-induced epithelial injury to downstream inflammatory and redox responses remain incompletely defined.

Interleukin-6 (IL-6), a pro-inflammatory cytokine, has been implicated in ocular surface inflammation. Elevated IL-6 levels are detected in tears of patients with DED [17, 18]. BAC exposure can stimulate IL-6 production in corneal epithelial cells [12, 19]. Cellular stress can also induce both IL-6 and soluble IL-6 receptor (sIL-6R) production in corneal epithelial cells [20, 21], suggesting that IL-6-dependent signaling may contribute to epithelial injury. IL-6 signaling occurs via distinct mechanisms, including cis-, trans-, and cluster signaling [22]. Cis-signaling occurs through membrane-bound IL-6 receptors (mIL-6R) and is generally associated with homeostatic, anti-inflammatory, and cytoprotective functions [23]. In contrast, IL-6 trans-signaling uses sIL-6R, formed by alternative splicing or proteolysis of mIL-6R, to drive pro-inflammatory responses [24]. IL-6 co-receptor, glycoprotein 130 (gp130), is required for signal transduction in both cis- and trans-signaling pathways [25]. Cluster signaling occurs when a transmitting cell presents IL-6/mIL-6R complexes to gp130 on adjacent cells. Accumulating evidence supports a role for IL-6 trans-signaling in cellular stress responses, redox imbalance, and barrier dysfunction across multiple cell types [20, 23, 26–29].

Soluble gp130 (sgp130) is a naturally occurring inhibitor of IL-6 trans-signaling. The fusion protein sgp130Fc consists of soluble gp130 linked to the Fc portion of human immunoglobulin G1 that promotes dimerization and improves binding efficiency [30, 31]. At low concentrations, sgp130Fc preferentially inhibits IL-6 trans-signaling without affecting cis-signaling by binding IL-6/sIL-6R complexes rather than IL-6 alone [30, 32]. We have previously shown that selective inhibition of IL-6 trans-signaling with sgp130Fc protects against inflammation, oxidative stress, mitochondrial dysfunction, barrier disruption, and apoptosis in human retinal endothelial cells [33, 34].

Although BAC-induced corneal epithelial toxicity is well established, the role of IL-6 trans-signaling in this response remains poorly understood. Therefore, this study investigated whether selective inhibition of IL-6 trans-signaling with sgp130Fc mitigates BAC-induced inflammatory activation, oxidative imbalance, endoplasmic reticulum (ER) stress, and epithelial barrier dysfunction. The findings of this study have shown that IL-6 trans-signaling might contribute to BAC-induced corneal epithelial injury in DED.

## Methods

### Cell culture and BAC treatment

Human telomerase-immortalized corneal epithelial (hTCEpi) cells [35] were cultured in serum-free keratinocyte growth medium-2 with keratinocyte growth medium-2 supplement kit and 0.15 mM calcium chloride at 37 °C and 5% CO_2,_ as previously described [36, 37]. Cells were seeded at 3×10^5^ cells per well in 12-well plates, allowed to adhere overnight, and maintained in basal media prior to treatment. hTCEpi cells were exposed to 0.001% BAC (#B6295, Sigma) for 30 min. To selectively inhibit IL-6 trans-signaling, cells were pretreated with 10 μg/mL sgp130Fc (#671-GP-100, R&D Systems) for 2 h prior to BAC exposure.

### Reverse transcriptase polymerase chain reaction (RT-PCR) analysis

RNA was isolated from hTCEpi cells by phenol–chloroform extraction using TRIzol reagent, and cDNA was synthesized with High-Capacity cDNA Reverse Transcriptase Kit (#4368814, Applied Biosystems). Primer sets were designed with NCBI Primer-BLAST and purchased from Integrated DNA Technologies. The primer sequences are shown in Table 1. RT-PCR was performed using 2X Universal SYBR Green Fast qPCR Mix (#RK21203, ABclonal) and a Bio-Rad CFX Connect Thermocycler. Gene expression was calculated relative to *GAPDH* using the 2^−ΔΔCq^ method, expressed as fold change relative to untreated cells.

**Table 1:** Primer sequences used for RT-PCR analysis

Gene	Forward primer (5ʹ → 3ʹ)	Reverse primer (5ʹ → 3ʹ)
*IL6*	CTCACCTCTTCAGAACGAATTGACAAACAAA	GGTACTCTAGGTATACCTCAAACTCCAAAA
*IL6RA*	GCTGTGCTCTTGGTGAGGAAGTTT	CTGAGCTCAAACCGTAGTCTGTAGAAA
*MCP1*	TCAAACTGAAGCTCGCACTCT	GGCATTGATTGCATCTGGC
*ICAM1*	AGGGTAAGGTTCTTGCCCAC	TGATGGGCAGTCAACAGCTA
*SOD1*	ACAAAGATGGTGTGGCCGAT	AACGACTTCCAGCGTTTCCT
*PRDX1*	GTTGGGACTGCTGATAGGAAG	AAGCACCAATCACTTGGCAG
*PRDX2*	ACCGAGATCATCGCGTTCAG	TAAAGAGGCCCCTGTAGGCA
*GPX1*	GTCGGTGTATGCCTTCTCGG	CAGGAGAGAAGGGCAGCTGAT
*GPX4*	GCCTTTGCCGCCTACTGA	GAAGCCCCGGTACTTGTCCAG
*TXN*	CAGACTCCAGCAGCCAAGAT	AGCAACATCATGAAAGAAAGGCT
*HSP72 (HSPA1A)*	TCAAGGGCAAGATCAGCGAG	CAGCAATCTTGGAAAGGCCCC
*GAPDH*	AATGAAGGGGTCATTGATGG	AAGGTGAAGGTCGGAGTCAA

*RT-PCR* = reverse transcriptase polymerase chain reaction

### Western blotting analysis

Cell lysates were prepared in 1X RIPA buffer (#AKR191, Cell Biolabs, Inc.) supplemented with protease and phosphatase inhibitors. Protein concentration was quantified using bicinchoninic acid assay (#23235, Thermo Scientific). 20–30 μg protein was resolved using 4%–15% SDS-PAGE gels (Bio-Rad) and transferred onto nitrocellulose membranes. Membranes were blocked with 5% non-fat milk in tris-buffered saline with 0.1% Tween-20 for 2 h at room temperature and incubated with primary antibodies overnight at 4 °C, followed by secondary antibodies for 1 h at room temperature. Protein bands were detected using ECL substrate, visualized on a Bio-Rad ChemiDoc Imaging System, quantified with ImageJ, and normalized to β-actin. Data were presented as fold change relative to untreated cells.

The following primary antibodies were used: PRDX (1:100, #sc-137222, Santa Cruz Biotechnology), Catalase (1:250, #sc-271803, Santa Cruz Biotechnology), BiP (1:1000, #A0241, ABclonal), PERK (1:1000, #5683, Cell Signaling), Ero1α (1:1000, #3264, Cell Signaling), β-catenin (1:500, #8480S, Cell Signaling), Occludin (1:200, #40–4700, Invitrogen), Claudin-1 (1:1000, #A2196, ABclonal), IL-6R (1:1000, #A1570, ABclonal), gp130 (1:100, #sc-655, Santa Cruz Biotechnology), and β-actin (1:10,000, #AC028, ABclonal).

### Enzyme-linked immunosorbent assay (ELISA)

Corneal epithelial cells were seeded in 12-well plates and treated as described above. Conditioned media was collected immediately prior to assaying. sIL-6R and sgp130 levels were measured using commercially available ELISA kits (#KE00732, Proteintech and #DY228, R&D Systems, respectively). sIL-6R and sgp130 concentrations (pg/mL) were calculated from the standard curve and reported as the ratio of sIL-6R to sgp130. Secreted IL-6 levels were measured using a commercially available ELISA kit with a sensitivity of 0.057 pg/mL, according to the manufacturer’s instructions (#ENZ-KIT178, Enzo). IL-6 concentration (pg/mL) was calculated from the standard curve.

### Detection of total ROS and hydrogen peroxide (H_2_O_2_) production

Total ROS levels were measured using a 2’,7’-dichlorofluorescein diacetate (DCFDA, #D6883, Sigma) assay. hTCEpi cells were seeded at 60,000 cells per well in 96-well plates and allowed to adhere overnight. Cells were then incubated with 25 μM DCFDA for 45 min at 37 °C, washed twice with Dulbecco’s phosphate-buffered saline and then treated as described above. As a positive control, 100 μM pyocyanin (#601522, Cayman Chemical) was used. Fluorescence intensity was measured at Ex/Em = 485/535 nm using a Synergy H1 microplate reader and expressed as fold change relative to untreated cells.

H_2_O_2_ levels were measured using a ROS-Glo™ H_2_O_2_ Assay (#G8820, Promega) with slight modifications to the manufacturer’s protocol. Cells were seeded at 30,000 cells per well in 96-well plates and allowed to adhere overnight. Cells were concurrently incubated with H_2_O_2_ substrate solution and treated as described above for 2.5 h at 37 °C. ROS-Glo detection reagent was then added, followed by incubation for an additional 2 h at room temperature. Luminescence was measured using a Synergy H1 microplate reader and expressed as fold change relative to untreated cells.

### Measurement of total antioxidant capacity

Total antioxidant capacity was measured using an antioxidant assay kit (#709001, Cayman Chemical) according to the manufacturer’s protocol. Following treatment, cells were harvested from 12-well plates and centrifuged at 2000 × g for 10 min at 4 °C. Cell lysates were prepared by sonication and centrifugation in 22 μL of 1X assay buffer. For the assay, 10 μL of standards or samples (diluted 1:50 in 1X assay buffer) were added to each well, followed by 10 μL of metmyoglobin and 150 μL of chromogen. Reactions were initiated by adding 40 μL of 441 μM H_2_O_2_ working solution and incubated at room temperature for 5 min. Absorbance was measured at 750 nm, and total antioxidant capacity (in mM Trolox equivalents) was calculated from the Trolox standard curve.

### Calcium flux analysis

Intracellular calcium levels in hTCEpi cells were measured using a Fluo-3 AM (#ALX-620-003, Enzo) fluorescent indicator. Cells were seeded at a density of 60,000 cells per well in 96-well plates and allowed to adhere overnight. Fluo-3 AM was loaded after treatment with BAC to avoid interactions between surfactants and acetoxymethyl esters. Following treatment, cells were incubated with 5 μM Fluo-3 AM for 30 min at 37 °C. Cells were washed three times with calcium-free HBSS and incubated for an additional 30 min to allow de-esterification. Cells were washed again, and fluorescence intensity was measured at Ex/Em = 490/528 nm. Relative Fluo-3 fluorescence was expressed as fold change relative to untreated cells.

### Measurement of epithelial barrier function

Trans-epithelial electrical resistance (TER) was measured in hTCEpi cells using electric cell-substrate impedance sensing (ECIS) with an ECIS Z-Theta instrument, as previously described [33, 36]. A 96-well ECIS array (96W1E+, Applied BioPhysics) was coated with 10 μM L-cysteine and 10 μg/mL fibronectin, and 70,000 cells per well were seeded and allowed to adhere overnight. Cells were treated as described above and maintained at 37 °C and 5% CO_2_ for the duration of the assay. Immediately following the addition of BAC, resistance readings were continuously recorded at 4000 Hz every 4 min for 60 min to assess epithelial barrier function. Resistance values were normalized to initial resistance and plotted as a function of time.

### Statistical analysis

Statistical analyses were performed using GraphPad Prism version 10.0 (GraphPad Software, Inc.) using one-way analysis of variance followed by Tukey’s or Holm-Šídák multiple comparisons test. *P* values < 0.05 were considered statistically significant. Data were expressed as mean ± standard error of the mean (SEM).

## Results

### BAC exposure activates IL-6 trans-signaling and downstream inflammatory responses in hTCEpi cells

**Fig. 1:**
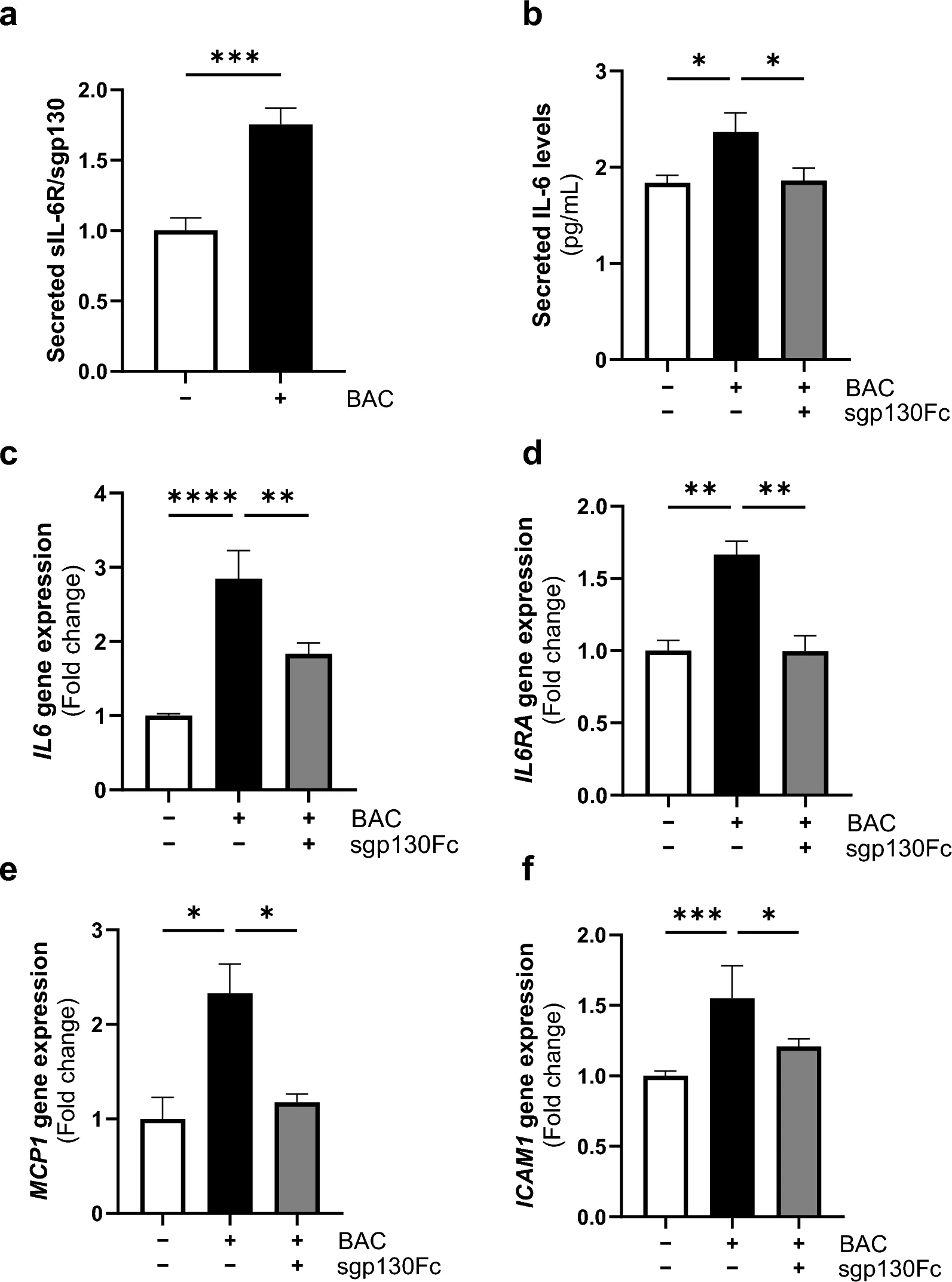
Benzalkonium chloride (BAC) exposure activates interleukin-6 (IL-6) trans-signaling and downstream inflammatory responses in human telomerase-immortalized corneal epithelial (hTCEpi) cells. **a**, **b** The sIL-6R/sgp130 ratio and secreted IL-6 protein levels were significantly increased in the conditioned media of BAC-treated hTCEpi cells as compared with untreated controls. **c**, **d** BAC-induced increases in *IL6* and *IL6RA* gene expression were significantly attenuated by selective inhibition of IL-6 trans-signaling with sgp130Fc, indicating partial protection against these effects. **e**,** f** Similarly, sgp130Fc partially prevented BAC-induced upregulation of monocyte chemoattractant protein 1 (*MCP1*) and intercellular adhesion molecule 1 *(ICAM1)* gene expression. Data are represented as mean ± standard error of the mean, n = 3–30/group; *P < 0.05, **P < 0.01, ***P < 0.001, ****P < 0.0001

To determine whether BAC promotes IL-6 trans-signaling, we first evaluated IL-6 signaling mediators in hTCEpi cells and conditioned media. BAC exposure significantly increased the sIL-6R/sgp130 ratio in conditioned media and secreted IL-6 protein levels (2.37 pg/mL vs. 1.83 pg/mL) compared with untreated controls (Fig. 1a, b). Simultaneously, BAC reduced intracellular mIL-6R protein levels (0.67-fold), whereas gp130 levels remained unchanged (Additional file 1). BAC also significantly increased *IL6* mRNA expression (2.85-fold) and *IL6RA* expression (1.67-fold), both of which were attenuated by sgp130Fc pretreatment (Fig. 1c, d). We next examined downstream inflammatory mediators of IL-6 and found that BAC significantly upregulated monocyte chemoattractant protein 1 (*MCP1*; 2.33-fold) and intercellular adhesion molecule 1 *(ICAM1*; 1.55-fold) mRNA levels, whereas sgp130Fc significantly attenuated these responses (Fig. 1e, f).

### sgp130Fc treatment mitigates BAC-induced oxidative imbalance in hTCEpi cells

**Fig. 2:**
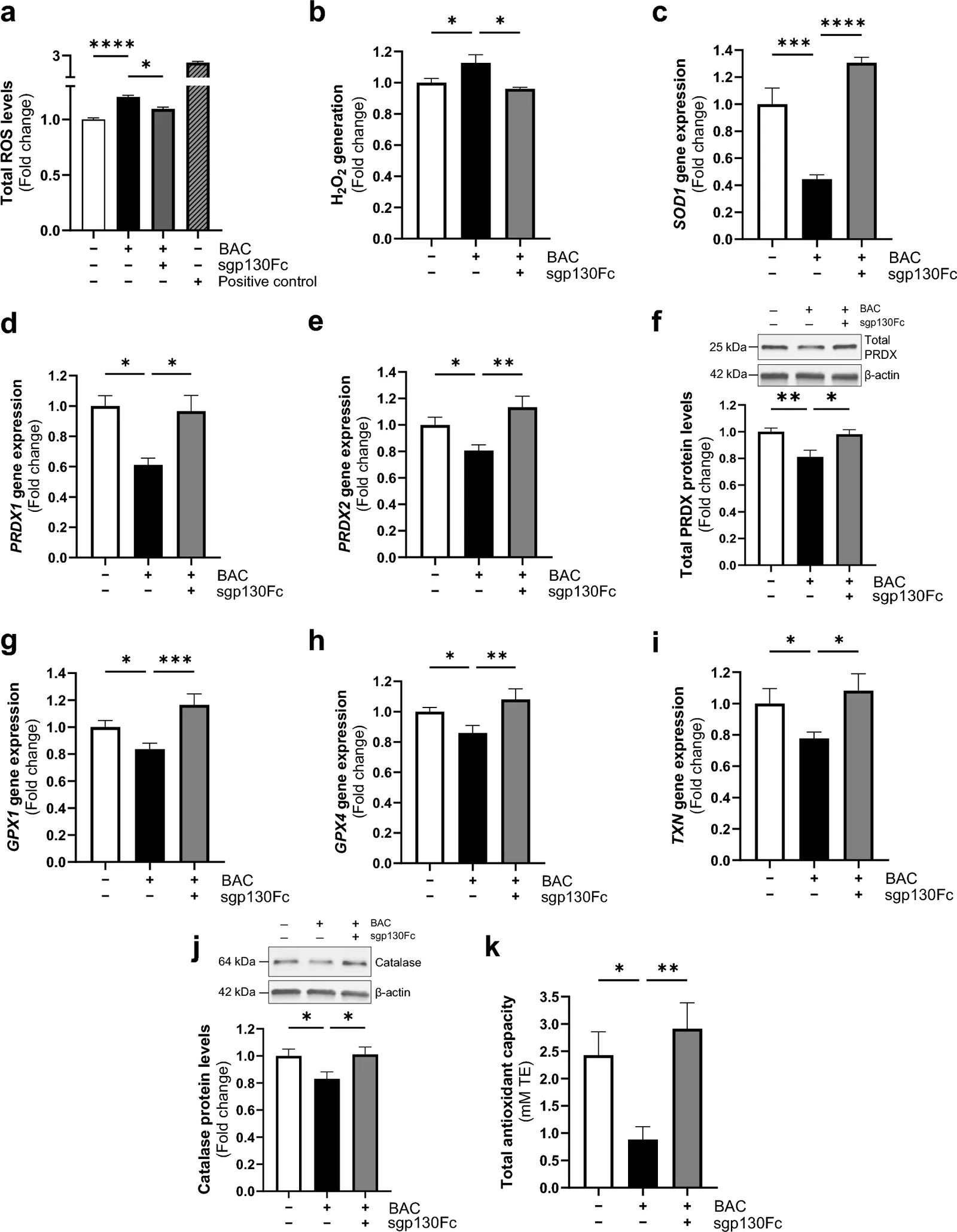
sgp130Fc treatment attenuates benzalkonium chloride (BAC)-mediated oxidative imbalance in human telomerase-immortalized corneal epithelial (hTCEpi) cells. **a** BAC-induced increases in total reactive oxygen species (ROS) levels were partially reduced by sgp130Fc treatment. 100 µM pyocyanin was used as a positive control. **b** Similarly, increased levels of hydrogen peroxide (H_2_O_2_) in BAC-treated hTCEpi cells were mitigated by sgp130Fc treatment. **c**–**f** sgp130Fc prevented the BAC-induced decrease in gene expression of several antioxidant enzymes (*SOD1*, *PRDX1*, and *PRDX2*) and reduction of total peroxiredoxin (PRDX) protein levels. β-actin is used as loading control. **g**–**j** A similar trend was observed for *GPX1*, *GPX4*, and *TXN* gene expression and catalase protein levels under these treatment conditions. β-actin was used as a loading control. **k** sgp130Fc treatment also restored total antioxidant capacity, measured in mM TE (Trolox equivalents), in BAC-treated hTCEpi cells. Data are represented as mean ± standard error of the mean, n = 3–23/group; *P < 0.05, **P < 0.01, ***P < 0.001, ****P < 0.0001

Oxidative stress occurs when ROS accumulation exceeds cellular antioxidant defenses. To evaluate whether BAC induces oxidative imbalance, total ROS and H₂O₂ levels were measured in hTCEpi cells. BAC exposure increased total ROS levels (1.18-fold), which were partially reduced by sgp130Fc pretreatment (Fig. 2a). Similarly, H₂O₂, which acts as a critical second messenger in redox signaling, was increased after BAC exposure (1.13-fold) but returned to baseline with sgp130Fc treatment (Fig. 2b). We next examined several antioxidant enzymes involved in redox homeostasis. Superoxide dismutase 1 (SOD1) is a major cytosolic enzyme that converts superoxide radicals into H₂O₂, whereas catalase, glutathione peroxidases (GPXs), peroxiredoxins (PRDXs), and thioredoxin (TXN) are key antioxidant enzymes involved in H₂O₂ detoxification. BAC treatment significantly downregulated *SOD1*, *PRDX1,* and *PRDX2* expression (0.44-fold, 0.61-fold, and 0.81-fold, respectively; Fig. 2c–e), with a corresponding decrease in total PRDX protein levels (0.81-fold; Fig. 2f). Pretreatment with sgp130Fc attenuated these BAC-induced reductions indicating that selective inhibition of IL-6 trans-signaling helps preserve antioxidant defenses during BAC-induced oxidative stress. BAC also reduced *GPX1*, *GPX4*, and *TXN* expression (0.84-fold, 0.86-fold, and 0.78-fold, respectively; Fig. 2g–i) and decreased catalase protein levels (0.83-fold; Fig. 2j). These changes were prevented by sgp130Fc pretreatment. Finally, BAC markedly impaired total antioxidant capacity by 64%, whereas inhibition of IL-6 trans-signaling with sgp130Fc maintained the antioxidant capacity at control levels (Fig. 2k).

### Inhibition of IL-6 trans-signaling protects against calcium flux and ER stress in BAC-treated hTCEpi cells

**Fig. 3:**
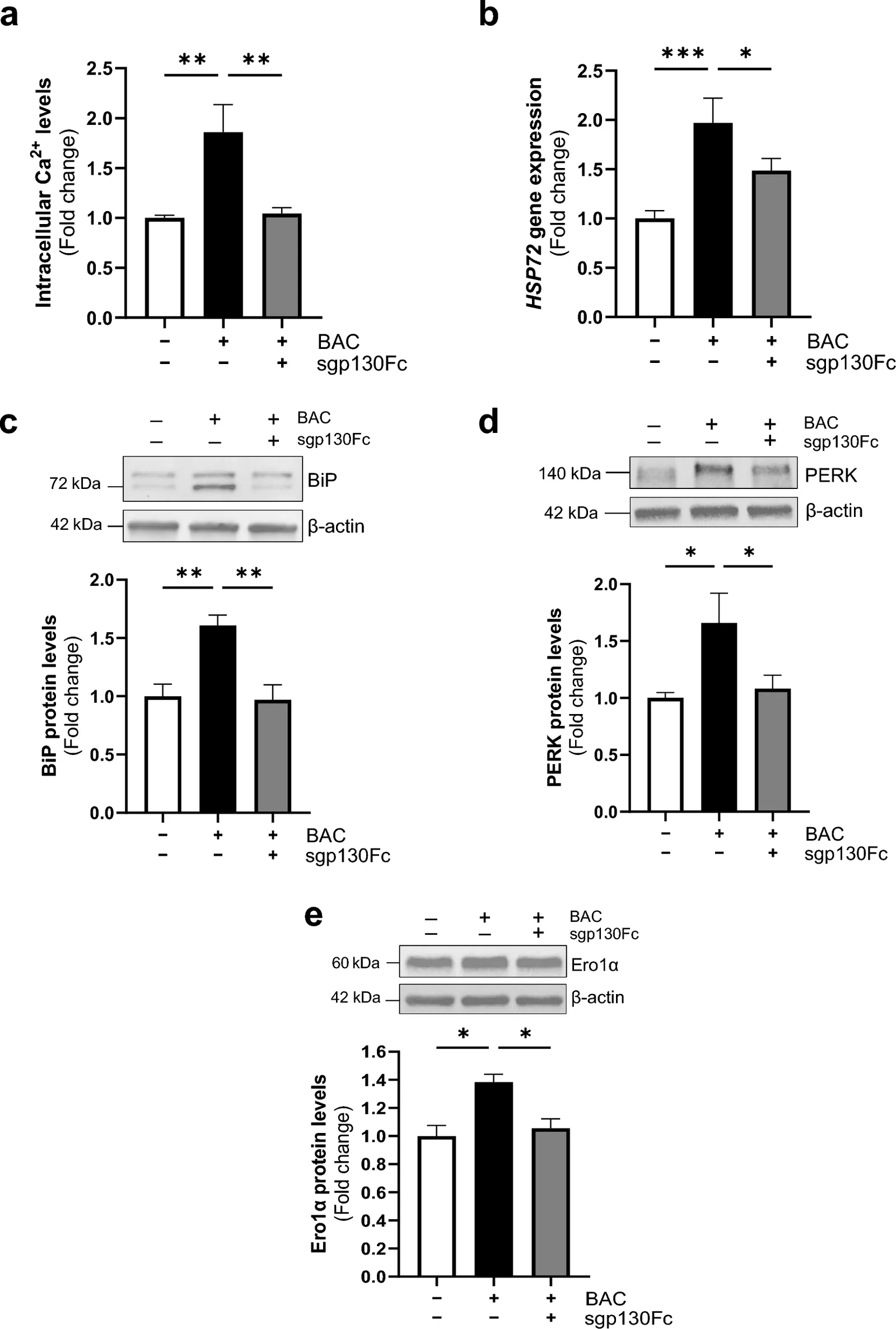
Interleukin-6 (IL-6) trans-signaling elevates intracellular calcium levels and endoplasmic reticulum (ER) stress in benzalkonium chloride (BAC)-treated human telomerase-immortalized corneal epithelial (hTCEpi) cells. **a** Intracellular Ca^2+^ levels were significantly elevated after BAC exposure, while sgp130Fc treatment had a protective effect against BAC-mediated Ca^2+^ flux. **b**, **c** Expression of cytosolic chaperone *HSP72* and levels of ER chaperone binding immunoglobulin protein (BiP) were significantly increased by BAC but attenuated with sgp130Fc treatment. **d**, **e** BAC exposure led to elevated levels of protein kinase RNA-like endoplasmic reticulum kinase (PERK), a sensor of ER stress, and Ero1α, a downstream target of PERK; however, blocking IL-6 trans-signaling with sgp130Fc mitigated BAC-induced upregulation of these ER stress mediators. β-actin was used as a loading control. Data are represented as mean ± standard error of the mean, n = 3–14/group; *P < 0.05, **P < 0.01, ***P < 0.001

To investigate BAC-mediated membrane destabilization and cellular stress responses, we assessed intracellular calcium levels and key markers of ER stress. BAC exposure significantly increased intracellular Ca^2^⁺ levels in hTCEpi cells (1.86-fold), whereas sgp130Fc pretreatment prevented BAC-induced Ca^2^⁺ flux (Fig. 3a). BAC also upregulated expression of heat shock protein 72 (*HSP72;* 1.97-fold), a cytosolic stress-response chaperone, and this increase was partially reduced by sgp130Fc pretreatment (Fig. 3b).

Similarly, protein levels of BiP, which is an ER chaperone and key regulator of ER homeostasis, were elevated following BAC exposure (1.61-fold) but returned to baseline levels after sgp130Fc pretreatment (Fig. 3c). BAC also upregulated the ER stress sensor protein kinase RNA-like endoplasmic reticulum kinase (PERK) by 1.66-fold and endoplasmic reticulum oxidoreductase 1 alpha (Ero1α), a downstream regulator of oxidative protein folding, by 1.38-fold. Pretreatment with sgp130Fc attenuated BAC-induced increases in PERK and Ero1α levels (Fig. 3d, e), suggesting that inhibition of IL-6 trans-signaling partially alleviates calcium dysregulation and ER stress in BAC-treated corneal epithelial cells.

### BAC-induced corneal epithelial barrier dysfunction is partially prevented by sgp130Fc

**Fig. 4:**
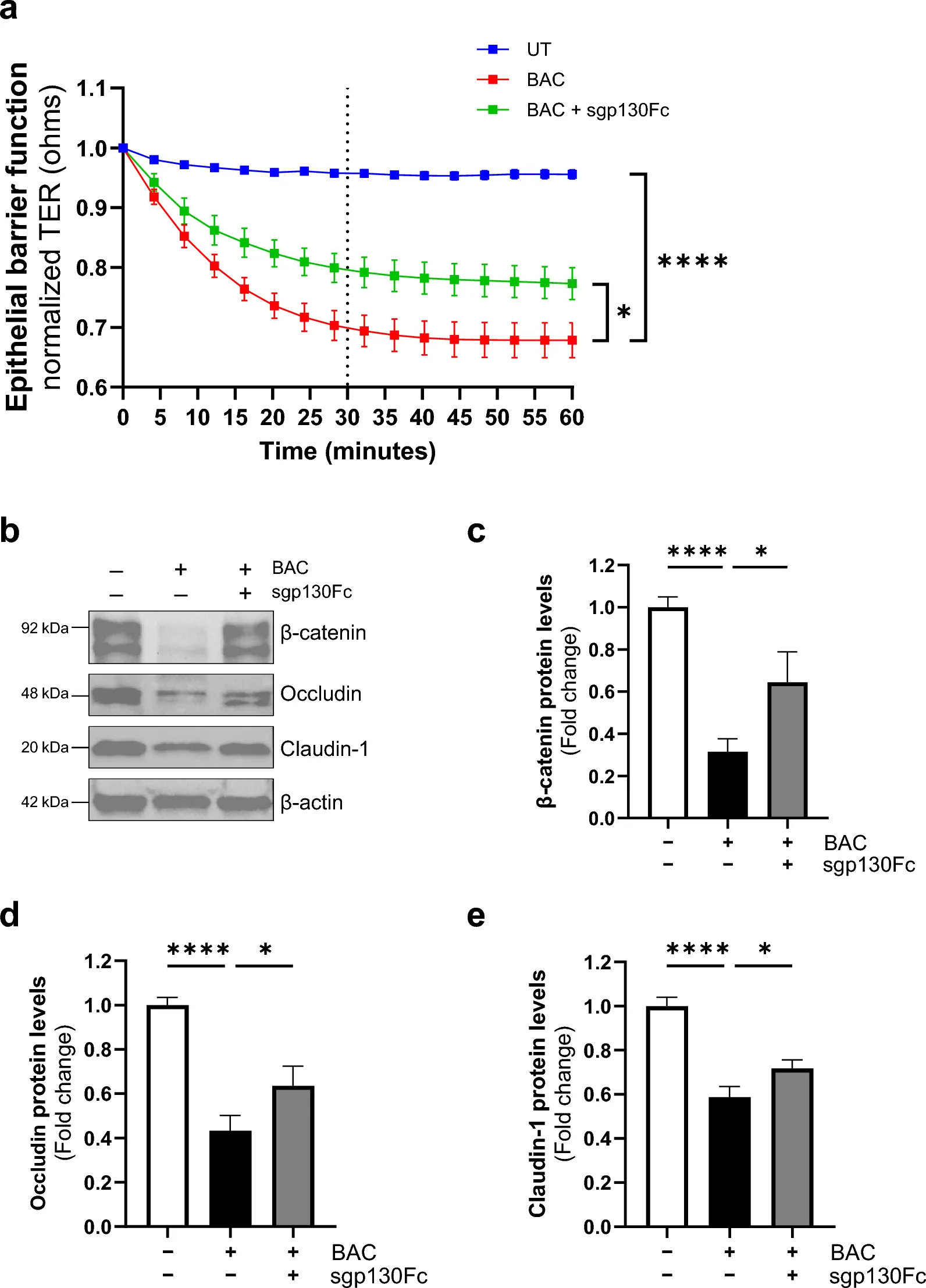
Benzalkonium chloride (BAC)-induced corneal epithelial barrier dysfunction is partially restored by sgp130Fc. **a** Trans-epithelial electrical resistance (TER) of human telomerase-immortalized corneal epithelial (hTCEpi) cell monolayers was monitored every 4 min for 60 min. BAC induced a progressive decline in TER reaching a 29% reduction at 60 min, whereas sgp130Fc partially prevented BAC-induced loss of barrier integrity. **b** Representative western blots of junctional proteins in hTCEpi cells. β-actin was used as a loading control. **c** Levels of β-catenin were significantly decreased with BAC treatment. **d**, **e** Levels of tight junction proteins, occludin and claudin-1, exhibited a similar decrease after BAC exposure. Inhibiting interleukin-6 trans-signaling with sgp130Fc showed partial protection against loss of these junctional proteins. Data are represented as mean ± standard error of the mean, n = 8–19/group; *P < 0.05, ****P < 0.0001

To assess whether inhibition of IL-6 trans-signaling preserves epithelial barrier integrity , BAC-exposed hTCEpi cells were analyzed by ECIS apparatus. BAC exposure caused a 29% reduction in TER after 60 min compared with untreated controls, indicating impaired epithelial barrier function. Pretreatment with sgp130Fc partially restored TER compared to the BAC-only group (Fig. 4a). We next evaluated adherens junction and tight junction proteins to determine whether changes in junctional protein levels contributed to BAC-induced barrier disruption. BAC exposure significantly reduced protein levels of β-catenin, occludin, and claudin-1 (0.31-fold, 0.43-fold, and 0.59-fold, respectively), whereas sgp130Fc pretreatment partially prevented the loss of these junctional proteins (Fig. 4b–e). These findings indicated that BAC compromises corneal epithelial barrier integrity and that selective inhibition of IL-6 trans-signaling partially preserves barrier function and junctional protein expression.

## Discussion

In DED, tear film instability and hyperosmolarity trigger cellular stress responses that promote the production of pro-inflammatory cytokines [1, 6, 38]. Elevated IL-6 levels have been detected in the tear fluid and conjunctival epithelium of patients with DED, which correlate with symptom severity and ocular surface parameters [17, 18, 39]. Experimental models further support a potential role for IL-6 signaling in DED pathogenesis. Both desiccating-stress and scopolamine-induced models of DED show a more pronounced IL-6 increase in ocular tissues compared to other cytokines [20, 40]. Moreover, tears from patients with DED contain elevated levels of IL-6 signaling receptors including sIL-6R, implicating these cytokine-receptor interactions as potential therapeutic targets for DED[41].

BAC is one of the most widely used preservatives in ophthalmic formulations, typically at concentrations ranging from approximately 0.004% to 0.02%. In addition to its bactericidal properties, BAC can increase epithelial permeability and enhance drug penetration [42]. However, clinical evidence indicates that the prevalence and severity of dry eye are associated with chronic use of topical eye drops [43–45]. Consequently, BAC is increasingly used in in-vitro models to study pro-inflammatory responses and investigate the molecular mechanisms underlying epithelial injury [19, 46, 47]. Notably, BAC exhibits dose- and time-dependent cytotoxic effects at concentrations lower than those used in many ophthalmic formulations [14]. Previous studies have demonstrated that short-term exposure to 0.001% BAC is sufficient to induce corneal epithelial dysfunction, including cytoskeletal alterations and membrane disruption, and has been shown to maintain adequate cell viability in mechanistic studies [14, 47–49]. These findings and our 3-(4,5-dimethylthiazol-2-yl)−2,5-diphenyltetrazolium bromide (MTT) viability assay (Additional file 2) support the use of this concentration as a sub-cytotoxic but biologically relevant injury model that mimics selected features of epithelial dysfunction observed in DED.

**Fig. 5:**
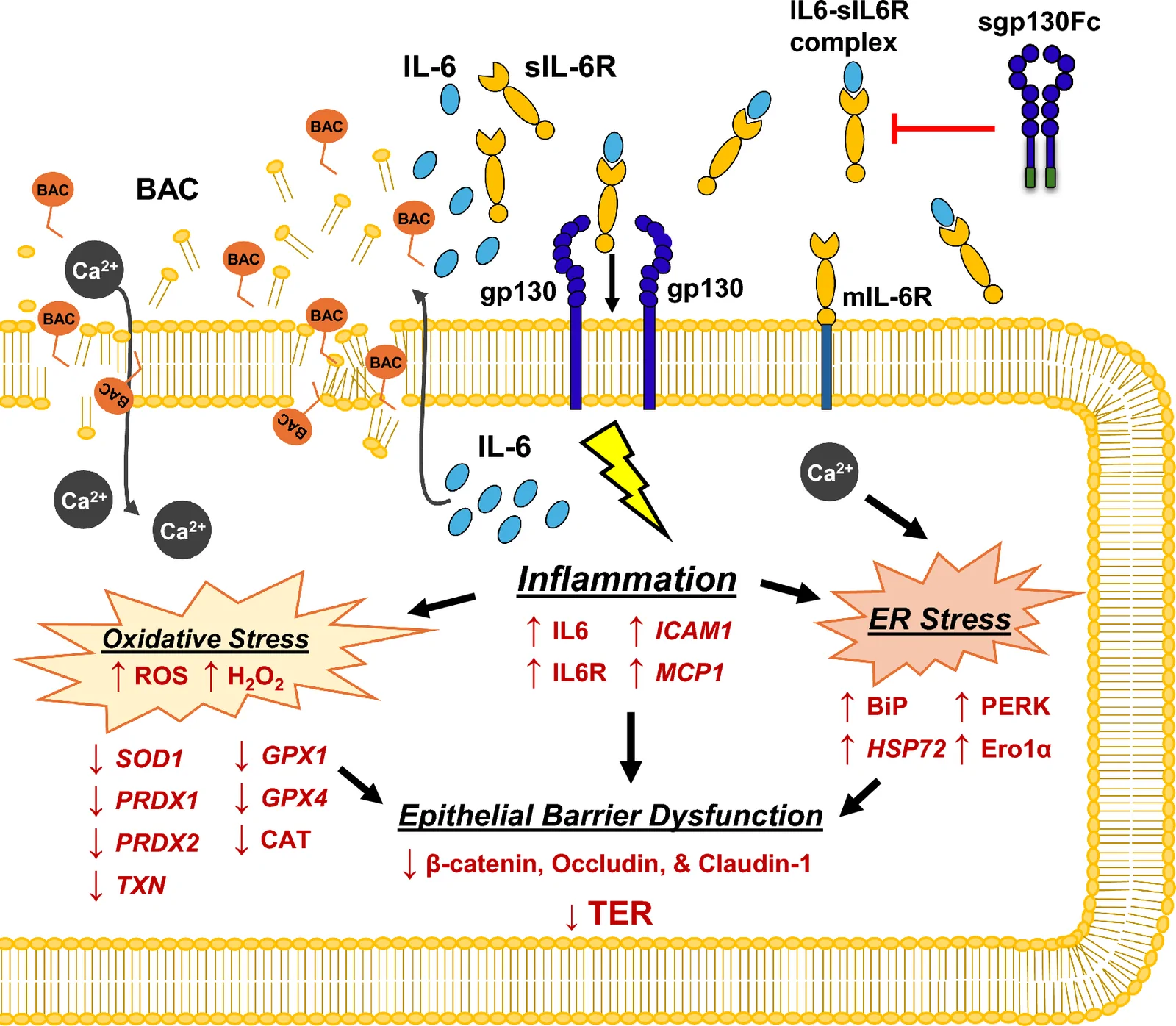
Proposed molecular mechanisms of interleukin-6 (IL-6) trans-signaling in benzalkonium chloride (BAC)-induced corneal epithelial dysfunction. BAC impairs corneal epithelial barrier integrity while promoting inflammation, oxidative imbalance, ER stress, and junctional protein loss partially through an IL-6 trans-signaling-dependent mechanism. Selective inhibition of IL-6 trans-signaling with sgp130Fc attenuates these pathological changes. BiP, binding immunoglobulin protein; CAT, catalase; Ero1α, endoplasmic reticulum oxidoreductase 1 alpha; GPX, glutathione peroxidase; HSP72, heat shock protein 72; ICAM1, intercellular adhesion molecule 1; MCP1, monocyte chemoattractant protein 1; mIL-6R, membrane-bound IL-6 receptor; PERK, protein kinase RNA-like endoplasmic reticulum kinase; PRDX, peroxiredoxin; ROS, reactive oxygen species; sIL-6R, soluble IL-6 receptor; SOD1, superoxide dismutase 1; TER, trans-epithelial electrical resistance; TXN, thioredoxin

In the current study, BAC exposure increased the sIL-6R/sgp130 ratio in hTCEpi cells suggesting enhanced IL-6 trans-signaling. Increased sIL-6R relative to the endogenous inhibitor sgp130 may enhance the availability of IL-6/sIL-6R complexes capable of activating gp130-expressing cells, thereby promoting pro-inflammatory signaling. This shift may contribute to BAC-induced epithelial injury and ocular surface inflammation, further supporting a contributory role for IL-6 trans-signaling in BAC-induced corneal epithelial damage (Fig. 5).

Our previously published studies have shown that IL-6 trans-signaling mediates inflammation and oxidative stress in retinal endothelial cells [33, 34, 50]. Importantly, ICAM-1 and MCP-1 are upregulated following activation of IL-6 trans-signaling in both human retinal endothelial cells and hTCEpi cells [23, 26, 34]. In the present study, BAC exposure induced a similar inflammatory phenotype in hTCEpi cells, which was attenuated by selective inhibition of IL-6 trans-signaling with sgp130Fc. ICAM-1 plays an important role in immunoinflammatory signaling and is elevated both locally and systemically in DED [51]. Lifitegrast, an anti-inflammatory drug approved for DED, exerts its therapeutic effects by blocking the interaction between lymphocyte function-associated antigen-1 and ICAM-1 [52]. In addition, MCP-1 is a key chemokine that regulates monocyte recruitment and is markedly elevated in hTCEpi cells under hyperosmolar stress [53].

Oxidative stress is central to the progression of DED, in which ROS accumulation initiates and amplifies inflammatory signaling [2]. Environmental irritants, tear film instability, and hyperosmolarity promote ROS production, whereas impaired antioxidant defenses exacerbate oxidative damage at the ocular surface [54]. The conjunctival epithelium of patients with DED shows significantly reduced protein levels of SOD, GPX, and CAT, with reductions corresponding to DED symptom severity [55]. Although we found that BAC modestly increased total ROS and H_2_O_2_ production in corneal epithelial cells, expression of important antioxidant enzymes, including *SOD1, PRDX, GPX, TXN*, and CAT, as well as total antioxidant capacity were significantly reduced. Together, these findings provide evidence of impaired redox homeostasis and suggest that BAC-induced oxidative imbalance primarily reflects compromised antioxidant defenses rather than marked ROS accumulation. Importantly, pretreatment with sgp130Fc attenuated these effects, suggesting that IL-6 trans-signaling partially contributed to BAC-induced oxidative injury and may represent a regulatory pathway linking inflammation and redox dysfunction in DED.

Lipid peroxidation is a major mechanism that causes oxidative damage. Elevated lipid peroxidation markers in the tears and the conjunctival cells of patients with DED suggest a pathological role for lipid damage in DED [56]. We recently showed that hyperosmolar stress induces lipid dysregulation and lipotoxicity in vitro [37]. Interestingly, disruption of antioxidant enzymes, such as SOD1 and GPX, has been shown to promote peroxidation of corneal epithelial membranes and generation of reactive aldehydes, including 4-hydroxynonenal and malondialdehyde [57]. Members of the PRDX family, particularly PRDX1, may also regulate lipid peroxidation in corneal cells [58].

Oxidative stress can further disrupt cellular homeostasis by inducing ER stress and activating the unfolded protein response (UPR) [59]. UPR is initiated by the accumulation of misfolded proteins and results in the dissociation of BiP from ER stress sensors [60]. Hyperosmolar stress and IL-6 signaling have been shown to induce ER stress through activation of PERK [27, 60, 61]. During severe ER stress, PERK suppresses protein translation yet increases expression of the pro-apoptotic transcription factor C/EBP homologous protein. C/EBP homologous protein regulates multiple downstream targets, including Ero1α, which facilitates oxidative protein folding and generates H₂O₂ as a byproduct. Importantly, H₂O₂ accumulation within the ER is regulated by PRDX4 [62]. In this study, BAC-treated hTCEpi cells showed elevated Ero1α levels and significantly reduced total PRDX levels, suggesting impaired ER redox homeostasis.

During UPR activation, BiP and other chaperone proteins are upregulated to restore ER homeostasis. Elevated BiP expression has been reported in the conjunctival epithelium of patients with DED and in corneal epithelial cells under hyperosmolar stress [63, 64]. Cytosolic chaperones including HSP72 are similarly induced to limit cellular injury and promote proper protein folding. In addition to its chaperone function, HSP72 interacts with UPR mediators such as inositol-requiring enzyme 1α and protects corneal epithelial cells against oxidative stress [65, 66]. Consistent with these observations, our findings demonstrate that BAC exposure increased HSP72, BiP, PERK, and Ero1α levels in corneal epithelial cells, supporting a role for UPR signaling in BAC-induced cytotoxicity.

Calcium is a key signaling molecule that regulates ER homeostasis. Cytosolic Ca^2^⁺ levels are tightly regulated under healthy conditions but can be altered by extracellular Ca^2^⁺ influx or Ca^2^⁺ release from ER stores. Activation of ER stress signaling pathways promotes Ca^2^⁺ release [67]; however, excessive Ca^2^⁺ influx can also trigger ER stress, further disrupting Ca^2^⁺ homeostasis [68]. In this study, we observed changes in cytosolic Ca^2^⁺ levels, although the relative contributions of extracellular Ca^2^⁺ influx and intracellular Ca^2^⁺ release remain unclear. Importantly, IL-6 exposure has been linked to increased ER stress and reduced Ca^2^⁺ storage [69, 70]. Similarly, hyperosmolarity-induced models of DED demonstrate time-dependent increases in intracellular Ca^2^⁺ levels in corneal epithelial cells [71]. Ca^2^⁺ signaling also plays a critical role in epithelial wound healing, with injured cells exhibiting waves of Ca^2^⁺ flux [72]. In our study, sgp130Fc mitigated BAC-induced increases in BiP, PERK, Ero1α, and intracellular Ca^2^⁺ levels suggesting that blockade of IL-6 trans-signaling may partially alleviate BAC-induced ER stress and associated Ca^2^⁺ dysregulation.

The corneal epithelial barrier provides both physical and immunological protection against environmental insults while maintaining corneal transparency. Tight junctions are the primary cell-to-cell contacts that regulate paracellular permeability, whereas adherens junctions facilitate tight junction formation and provide structural support to maintain epithelial integrity [7]. In addition, epithelial barrier function depends on Ca^2^⁺ homeostasis and limits the penetration of both hydrophilic and hydrophobic ophthalmic drugs [73]. BAC disrupts corneal epithelial barrier function in a dose-dependent manner [74]. At concentrations at or above its critical micellar concentration of approximately 0.01%–0.02%, BAC can directly solubilize cellular membranes through its surfactant properties [14, 75]. In contrast, at sub-critical micellar concentrations, BAC can induce more gradual membrane destabilization likely involving inflammatory signaling and oxidative damage. Consistent with these effects, decreases in TER have been observed at BAC concentrations as low as 0.0001% with saturation effects occurring near 0.01% BAC [14]. BAC can also induce concentration-dependent leak currents across corneal epithelial membranes suggesting compromised barrier function and increased ion permeability [15]. Inflammatory and oxidative pathways may further amplify BAC-induced barrier disruption. IL-6 exposure has been shown to reduce TER in corneal epithelial cells, accompanied by alterations in junctional protein levels [3]. Similarly, ROS accumulation can increase paracellular permeability across epithelial tissues by exacerbating inflammatory injury and directly damaging junctional proteins [7].

Overall, our findings demonstrated that selective inhibition of IL-6 trans-signaling with sgp130Fc attenuates multiple BAC-induced injury responses including inflammatory activation, oxidative imbalance, ER stress, calcium dysregulation, and epithelial barrier disruption. These results support a contributory role for IL-6 trans-signaling in BAC-induced corneal epithelial injury and highlight sgp130Fc as a potentially promising approach for modulating ocular surface inflammation. However, several limitations should be considered when interpreting these findings. In this study, IL-6 secretion in hTCEpi cells exposed to 0.001% BAC for 30 min was modest, which is consistent with previous reports showing that these cells exhibited a limited secretory response compared with other cell types [76]. In addition, a partial rescue of key endpoints, including TER and junctional protein levels, indicated that additional IL-6-independent mechanisms, such as direct surfactant-mediated BAC effects and other stress pathways, also contributed to BAC-induced injury. The kinetics of BAC-induced IL-6 secretion and the reproducibility of these effects under additional inflammatory or chronic stress conditions require further investigation. Additionally, administration of sgp130Fc during or after BAC exposure may provide further therapeutic relevance in future studies. Since corneal epithelial cells have been reported to express Fc receptors [77], future studies should also include sgp130Fc-only and Fc-control groups to exclude potential Fc-mediated effects. Finally, although BAC exposure reproduces several features associated with dry eye, including inflammatory signaling and epithelial barrier disruption, it does not fully recapitulate the complex pathophysiology of DED. Therefore, these findings should be interpreted in the context of BAC-induced epithelial injury relevant to ocular surface dysfunction in DED.

## Conclusions

These findings suggest that BAC exposure activates pro-inflammatory IL-6 trans-signaling in hTCEpi cells. Selective inhibition of IL-6 trans-signaling with sgp130Fc attenuates inflammatory, oxidative, and stress responses while partially preserving epithelial barrier function. Targeting this pathway may represent a therapeutic strategy for reducing ocular surface inflammation and improving outcomes in DED.

## Supplementary Information

Additional file 1. Endogenous levels of membrane-bound IL-6 receptor and glycoprotein 130 in untreated and benzalkonium chloride-treated human telomerase-immortalized corneal epithelial cells. 

Additional file 2. Cell viability of human telomerase-immortalized corneal epithelial cells after 30-min exposure to different doses of benzalkonium chloride. 

Additional file 3. Raw data of western blot figures.

## Data Availability

All data generated or analyzed during this study are included in this article.

## Abbreviations

DCFDA: 2’,7’-Dichlorofluorescein diacetate
MTT: 3-(4,5-Dimethylthiazol-2-yl)− 2,5-diphenyltetrazolium bromide
BAC: Benzalkonium chloride
BiP: Binding immunoglobulin protein
CAT: Catalase
DED: Dry eye disease
ECIS: Electric cell-substrate impedance sensing
ER: Endoplasmic reticulum
Ero1α: Endoplasmic reticulum oxidoreductase 1 alpha
ELISA: Enzyme-linked immunosorbent assay
GPXs: Glutathione peroxidases
gp130: Glycoprotein 130
HSP72: Heat shock protein 72
hTCEpi: Human telomerase-immortalized corneal epithelial
H_2_O_2_: Hydrogen peroxide
ICAM1: Intercellular adhesion molecule 1
IL-6: Interleukin-6
mIL-6R: Membrane-bound IL-6 receptor
MCP1: Monocyte chemoattractant protein 1
PRDXs: Peroxiredoxins
PERK: Protein kinase RNA-like endoplasmic reticulum kinase
ROS: Reactive oxygen species
sIL-6R: Soluble IL-6 receptor
SEM: Standard error of the mean
SOD1: Superoxide dismutase 1
TXN: Thioredoxin
TER: Trans-epithelial electrical resistance
