# Supplementary material for: Blocking IL-6 trans-signaling mitigates benzalkonium chloride-induced inflammation, oxidative imbalance, ER stress, and barrier disruption in human corneal epithelial cells
Source: Eye Vis (Lond). 2026 Oct 20;13:46. doi: 10.1186/s40662-026-00513-0 (PMC13681774; doi:10.1186/s40662-026-00513-0)

Raw data of Figure 2f (Total PRDX)

Gel 164 (JG)

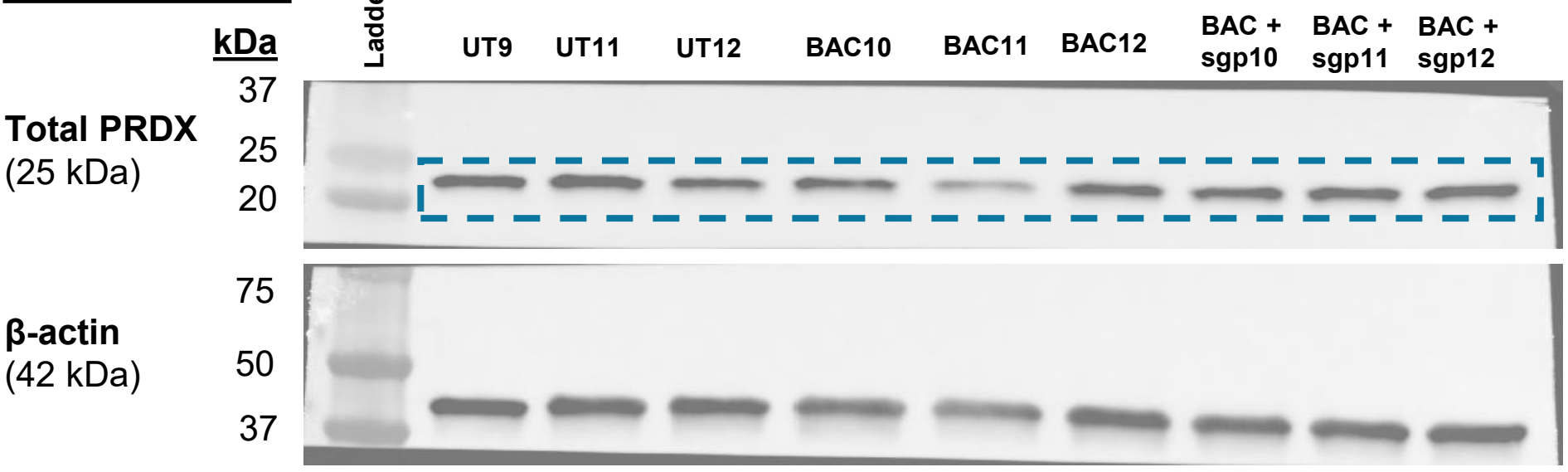

Gel 165 (JG)

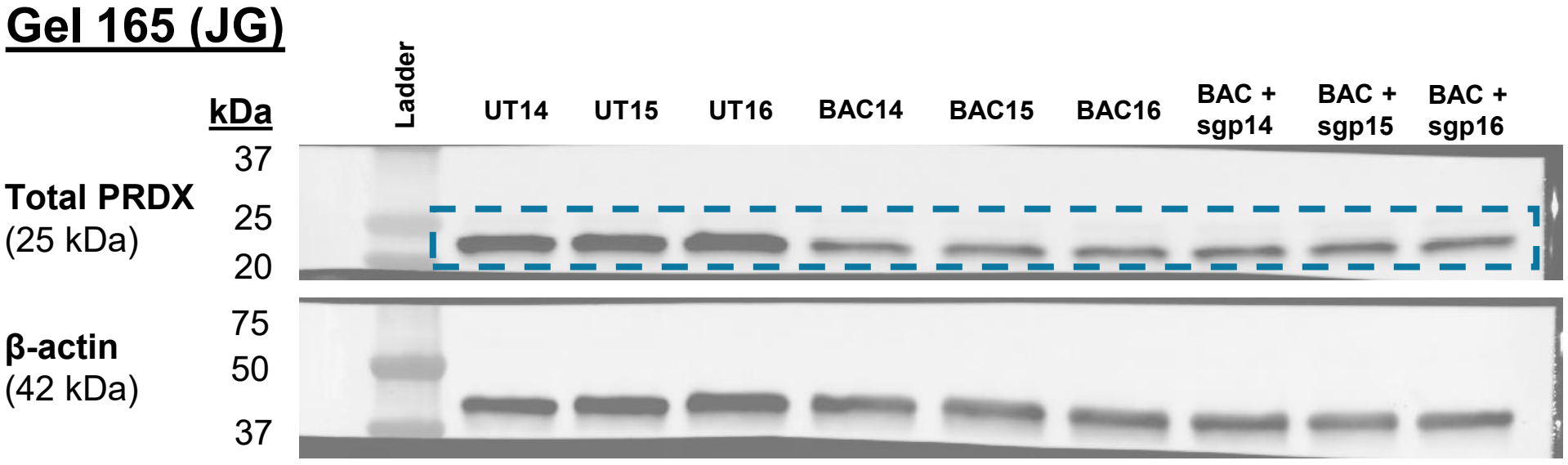

Gel 172 (JG)

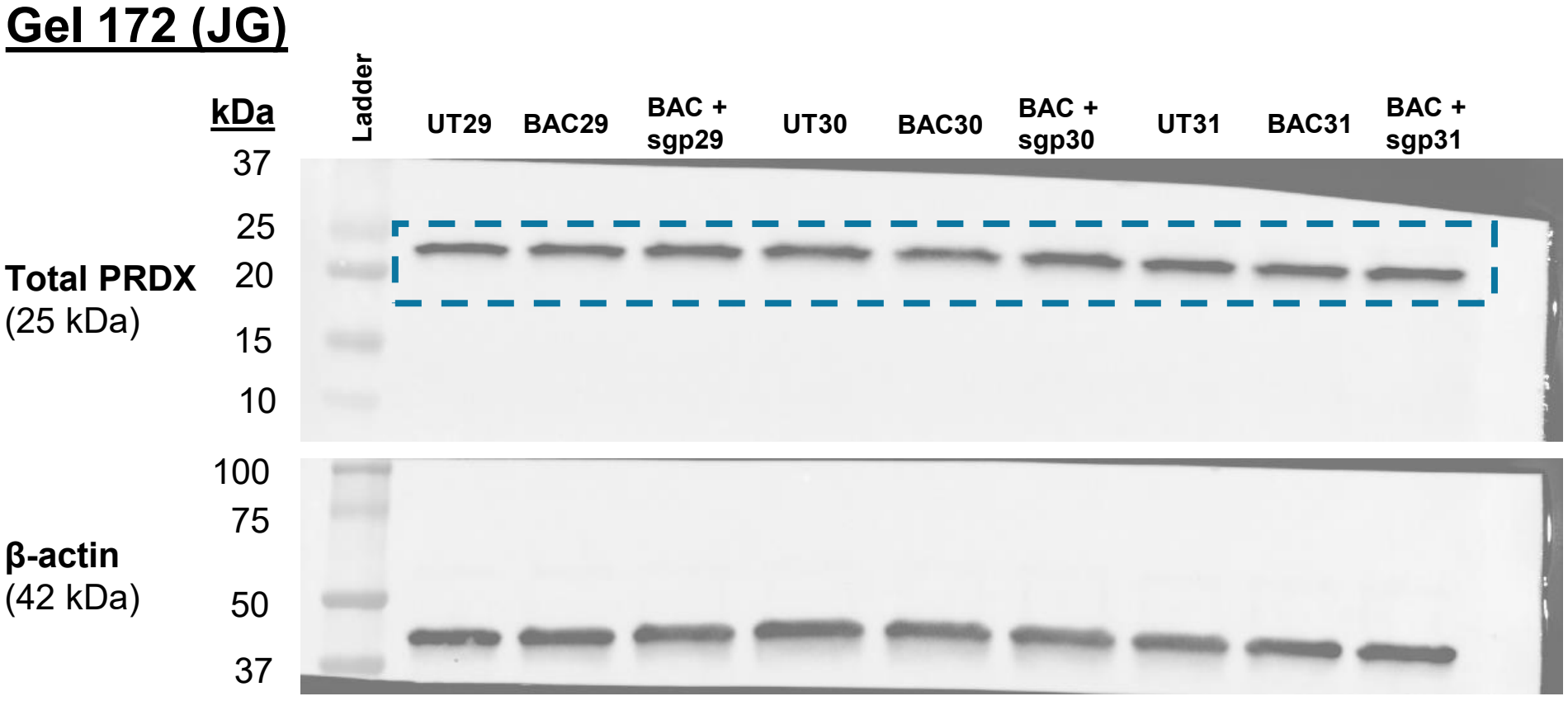

Gel 173 (JG)

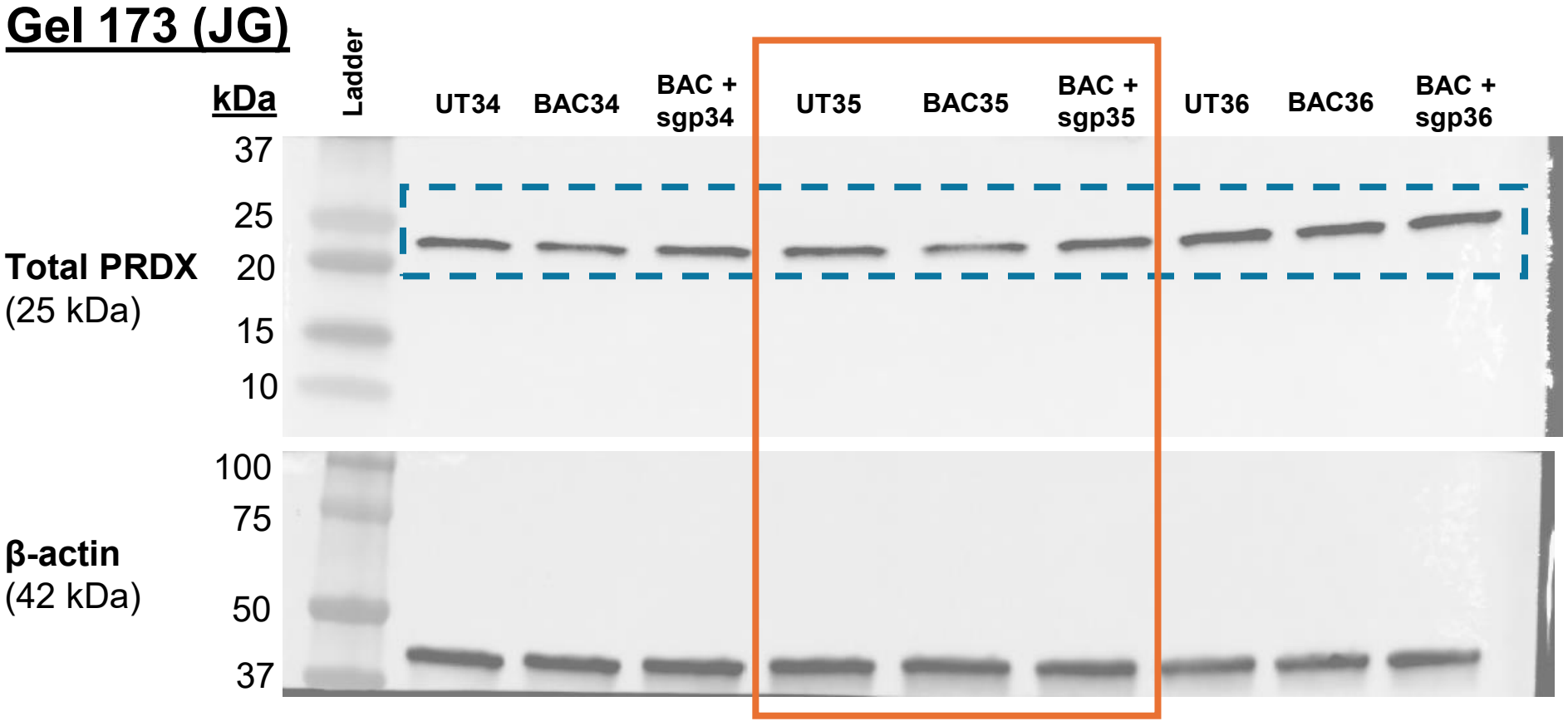

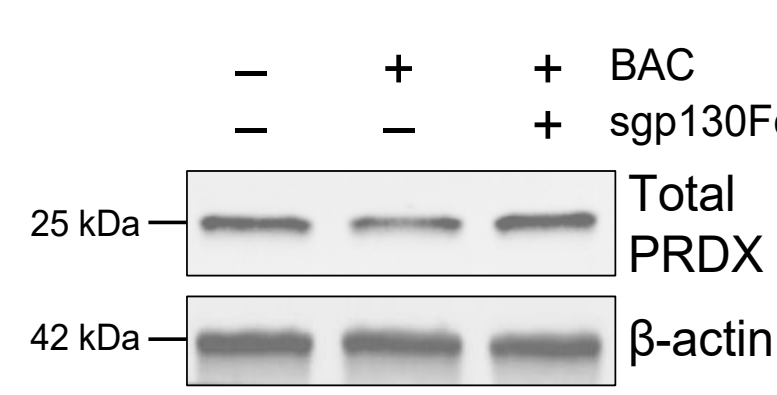

Used as representative for Figure 2F.

Raw data of Figure 2j (Catalase)

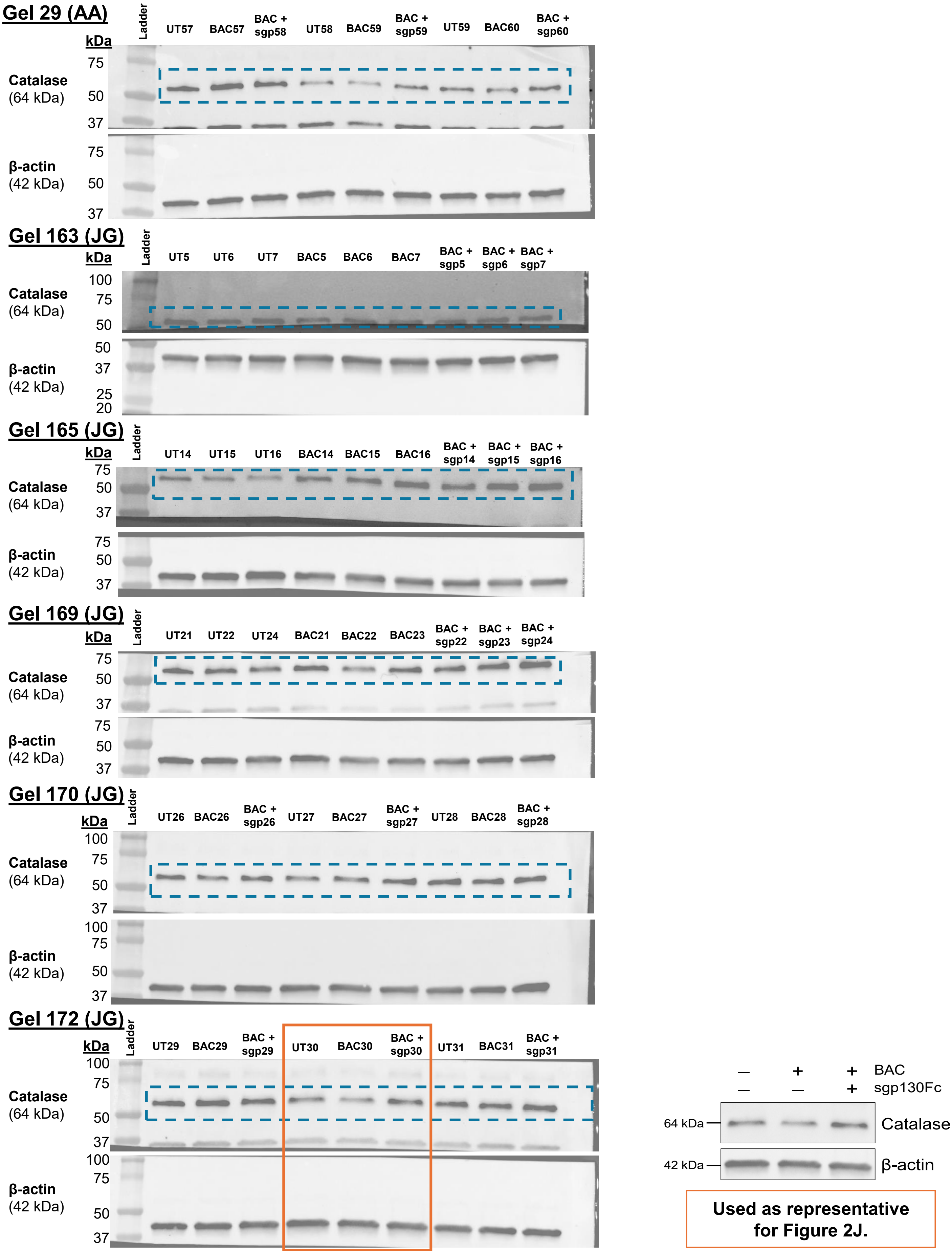

Raw data of Figure 3c (BiP)

Gel 166 (JG)

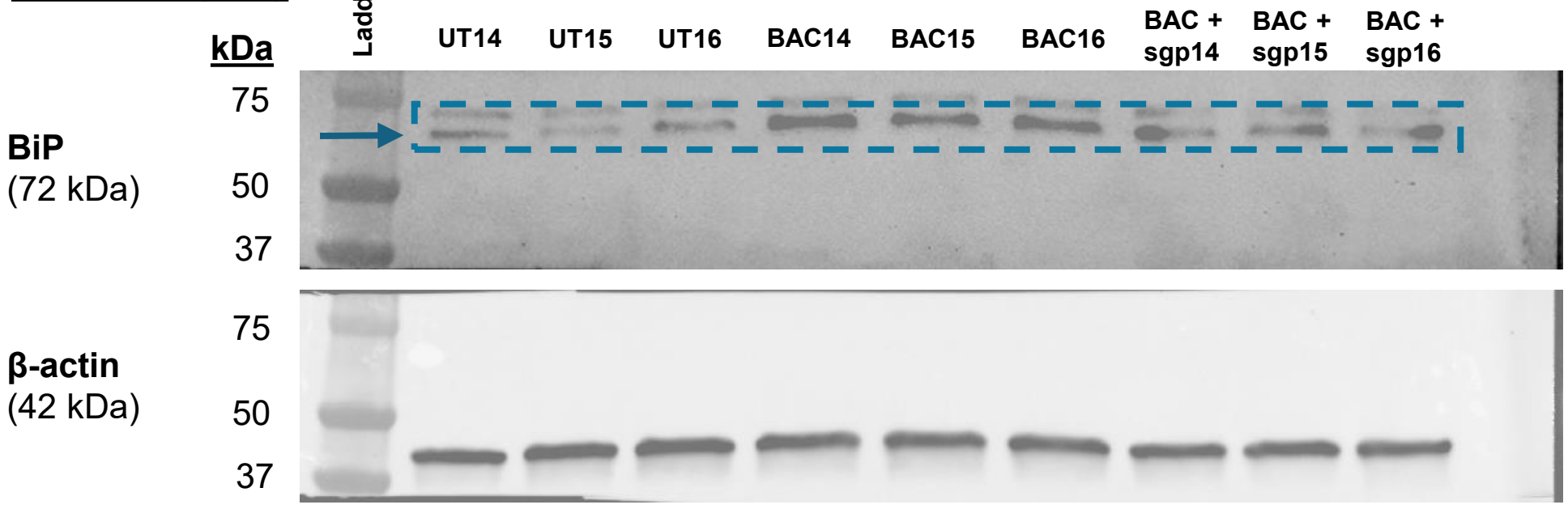

Gel 171 (JG)

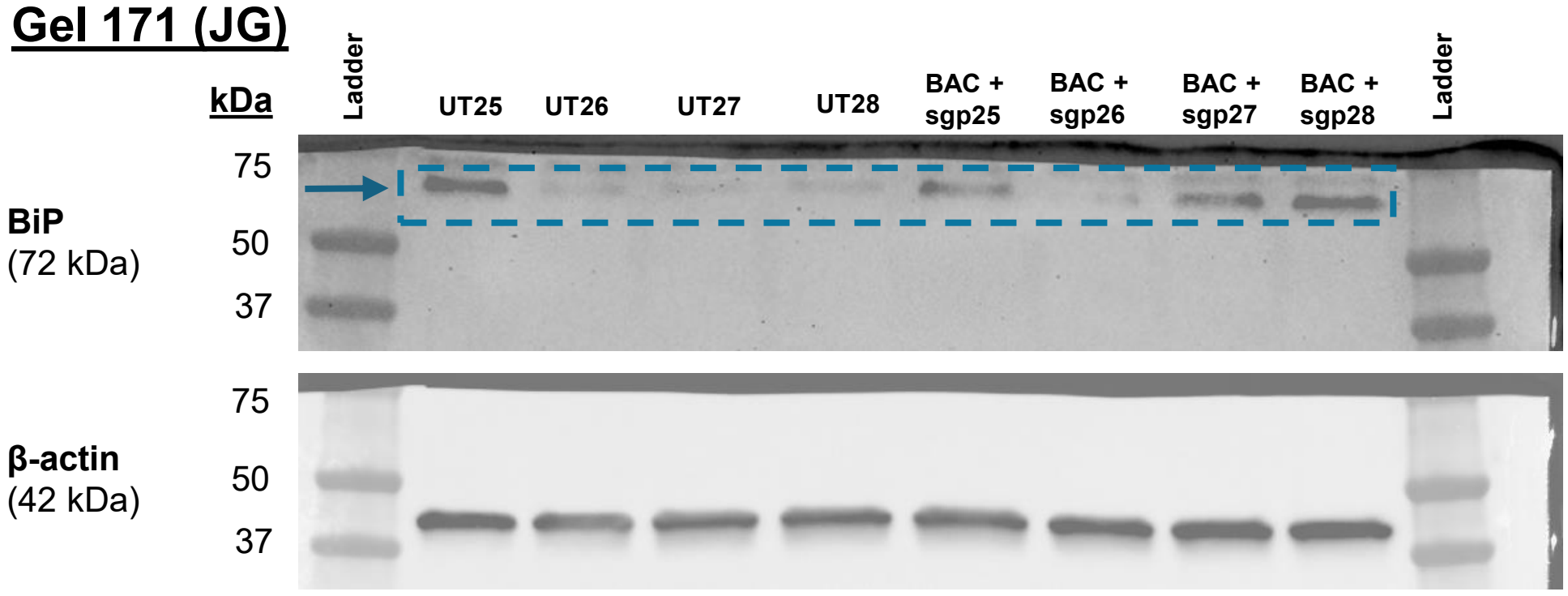

Gel 173 (JG)

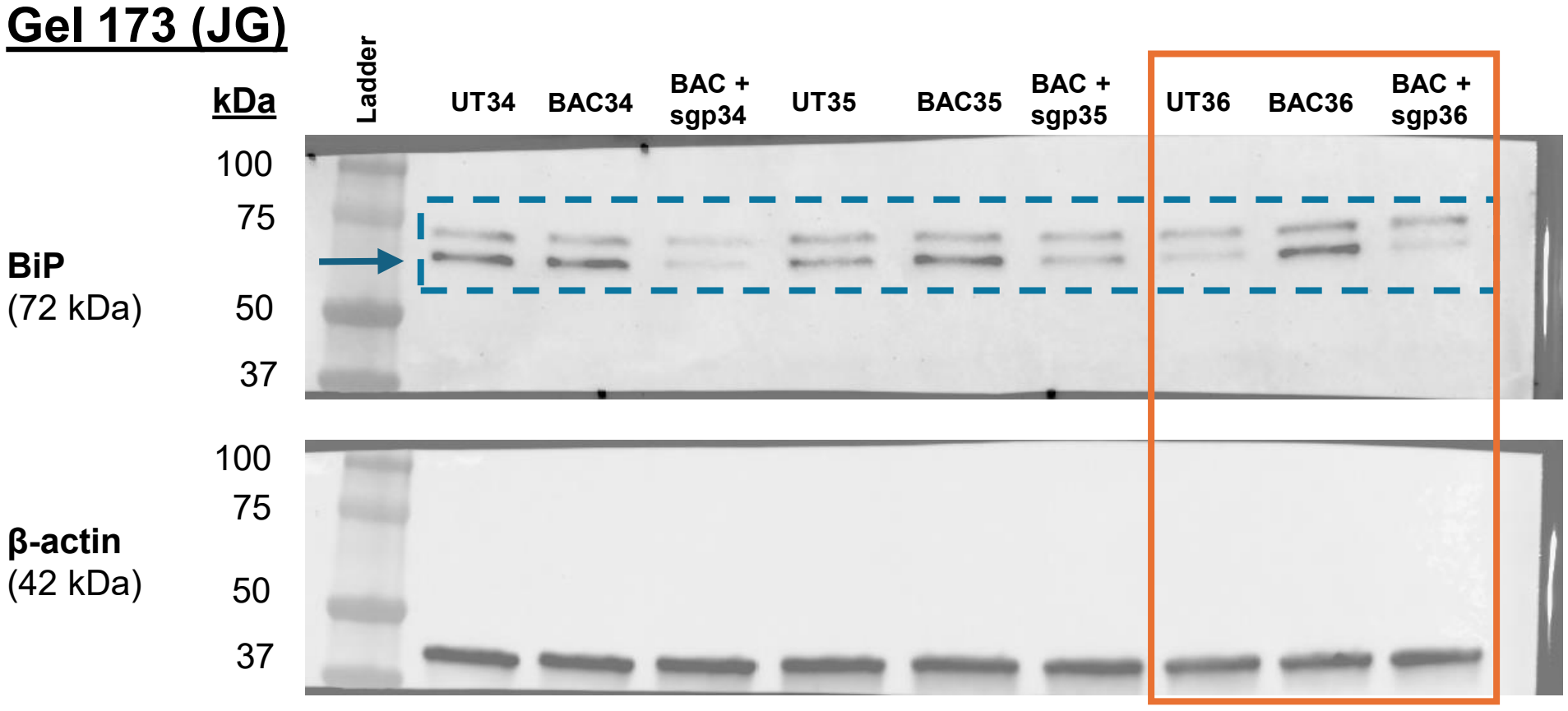

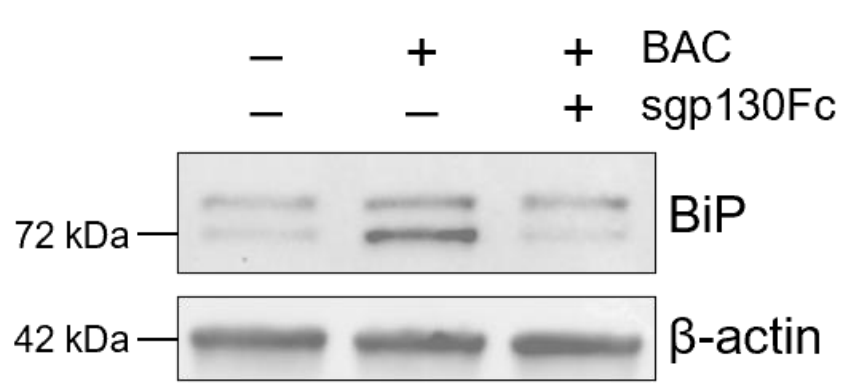

Used as representative for Figure 3C.

Raw data of Figure 3d (PERK)

Gel 21 (KL)

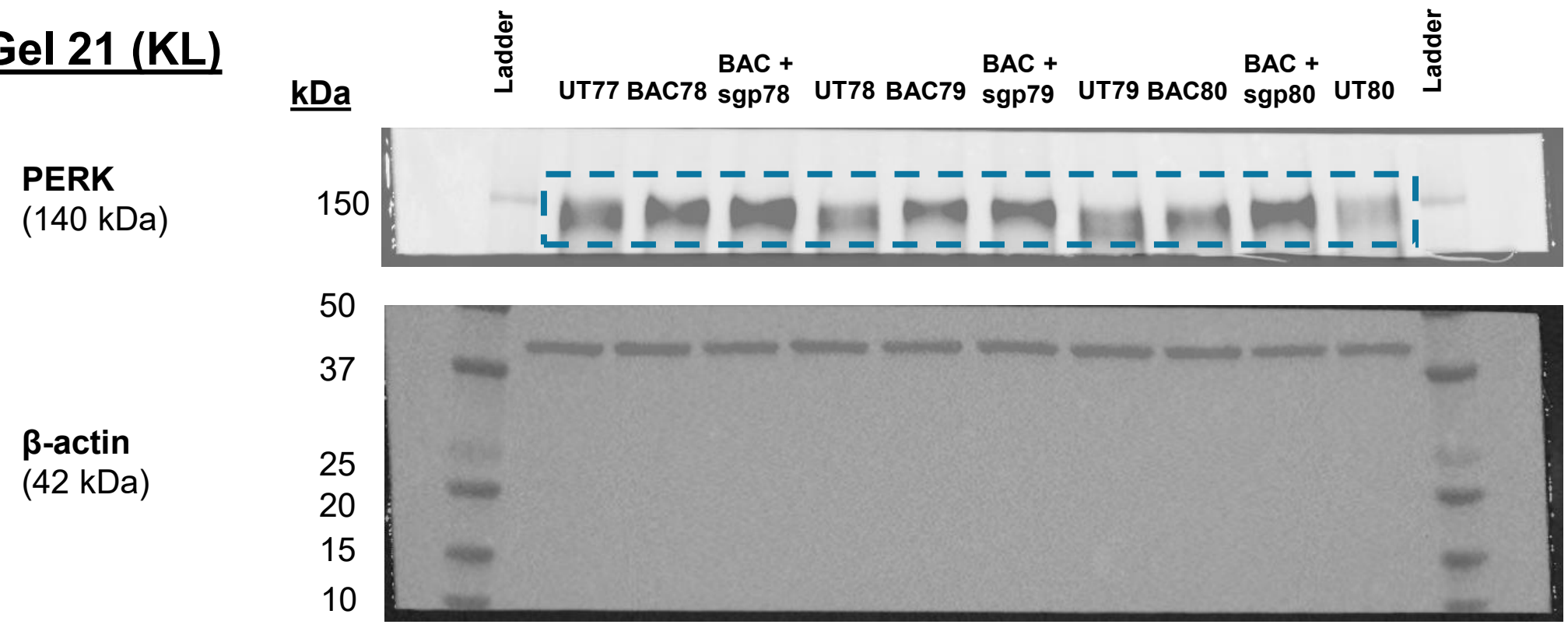

Gel 22 (KL)

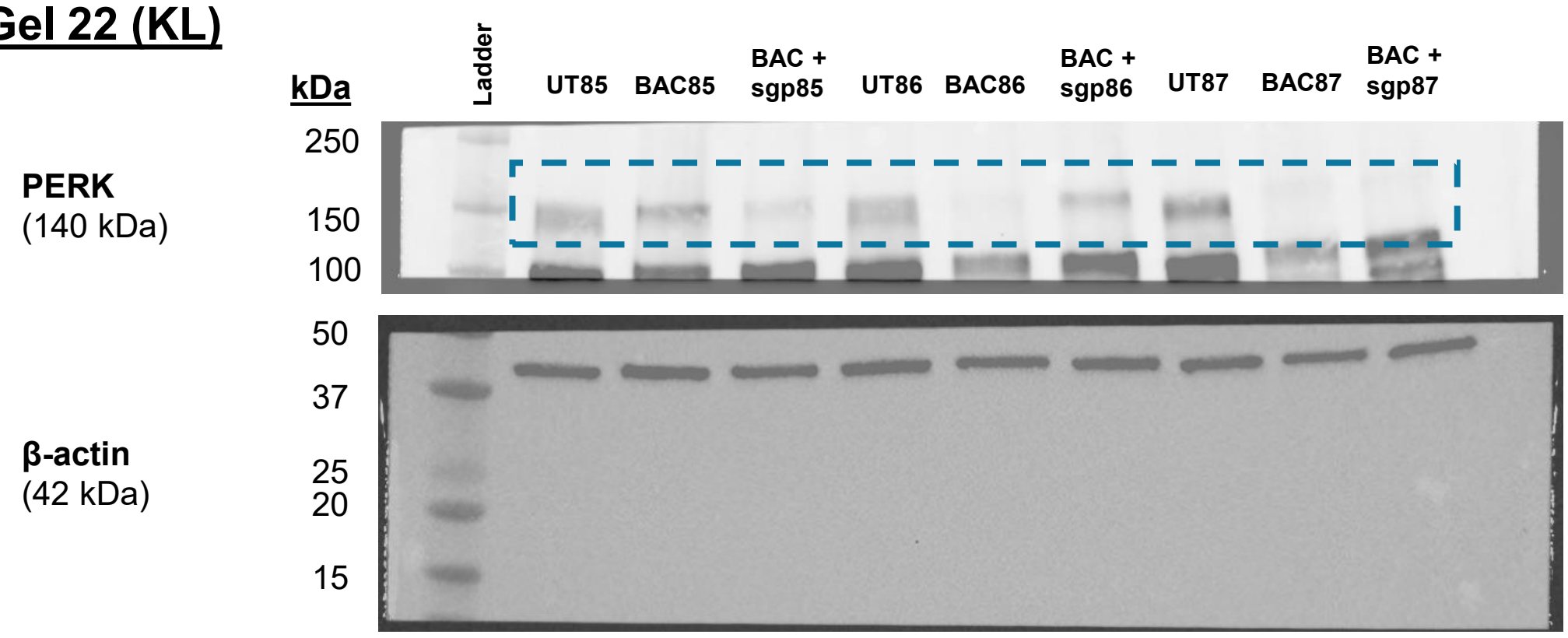

Gel 23 (KL)

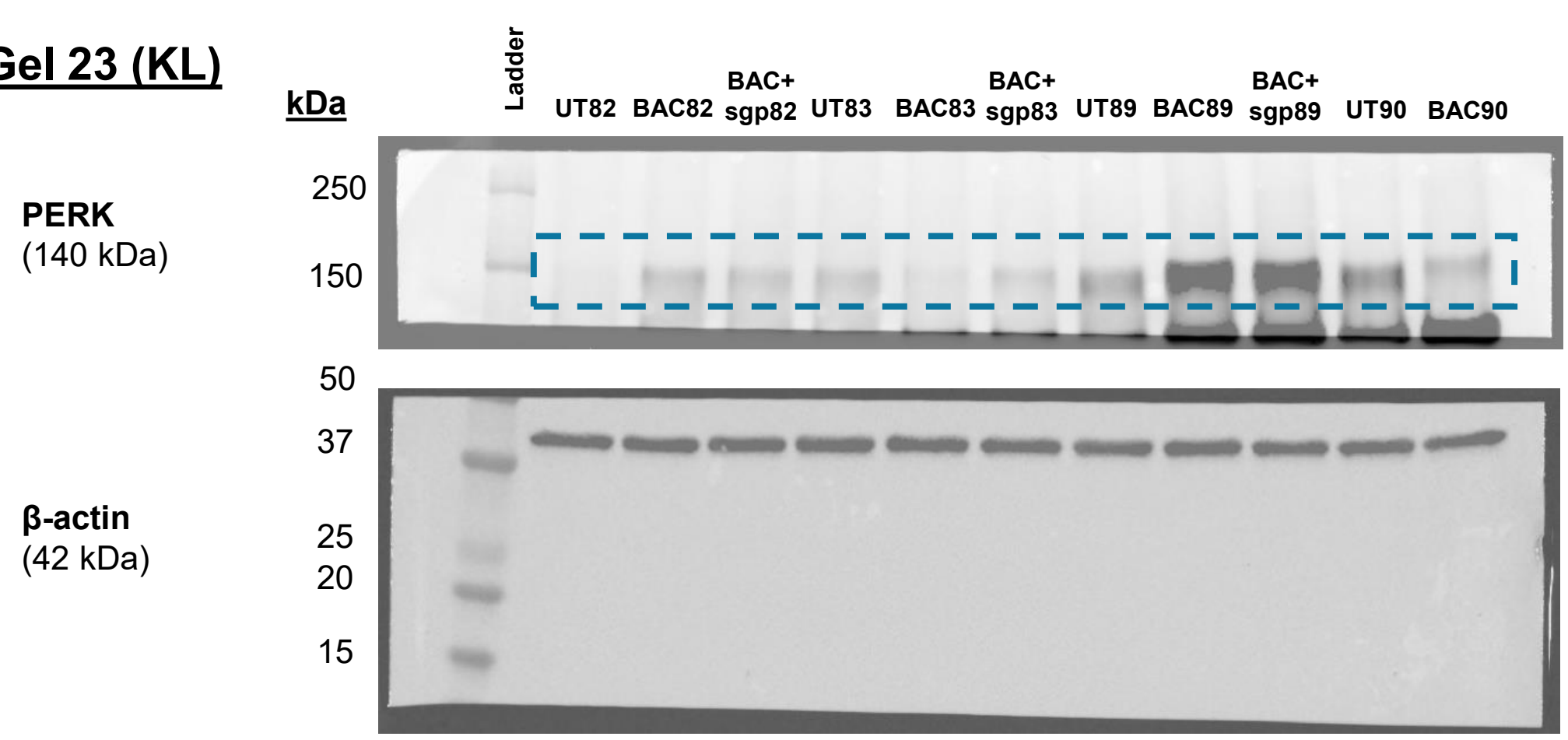

Raw data of Figure 3d (PERK) Continued

Gel 34 (AA)

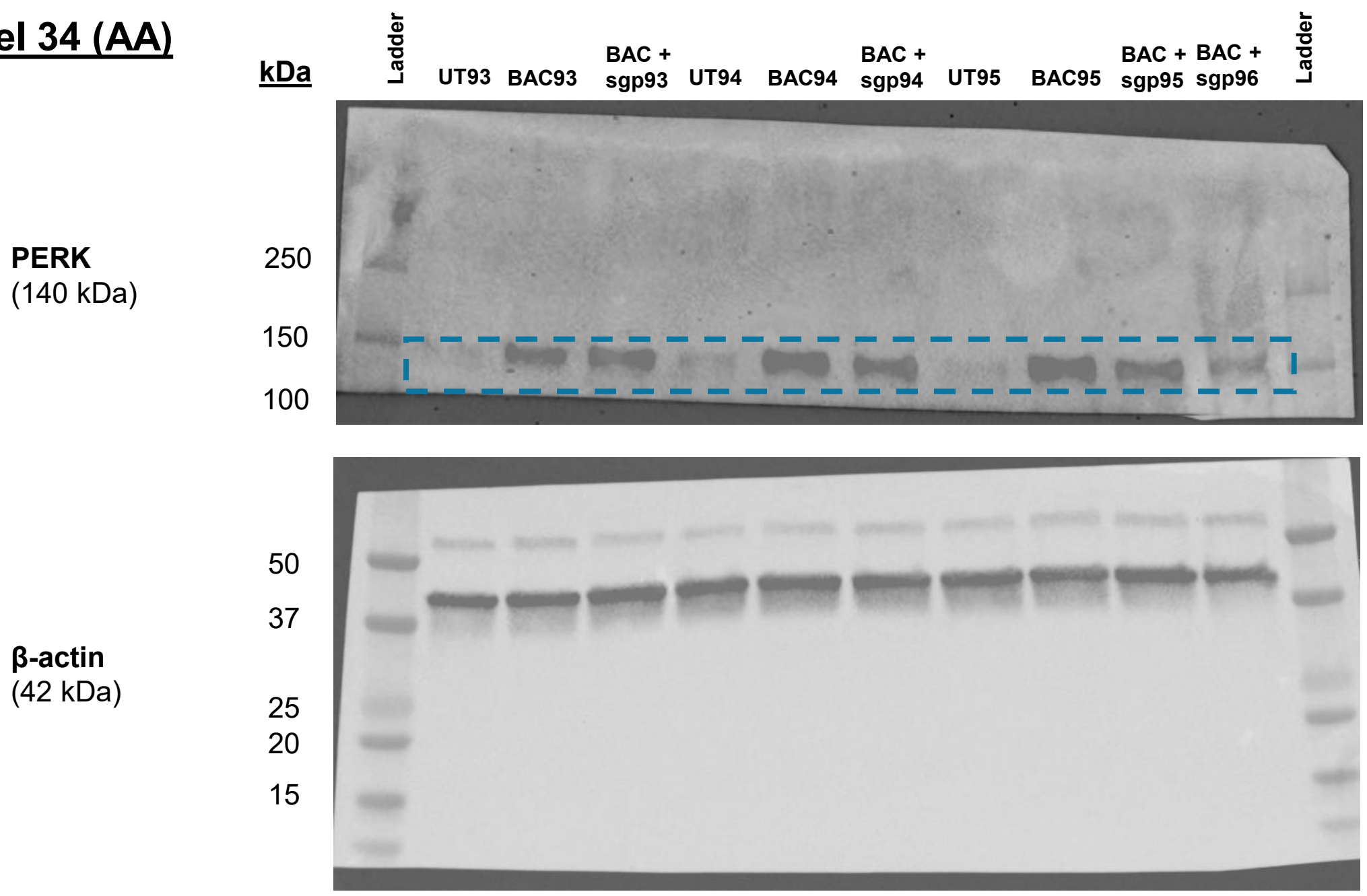

Gel 35 (AA)

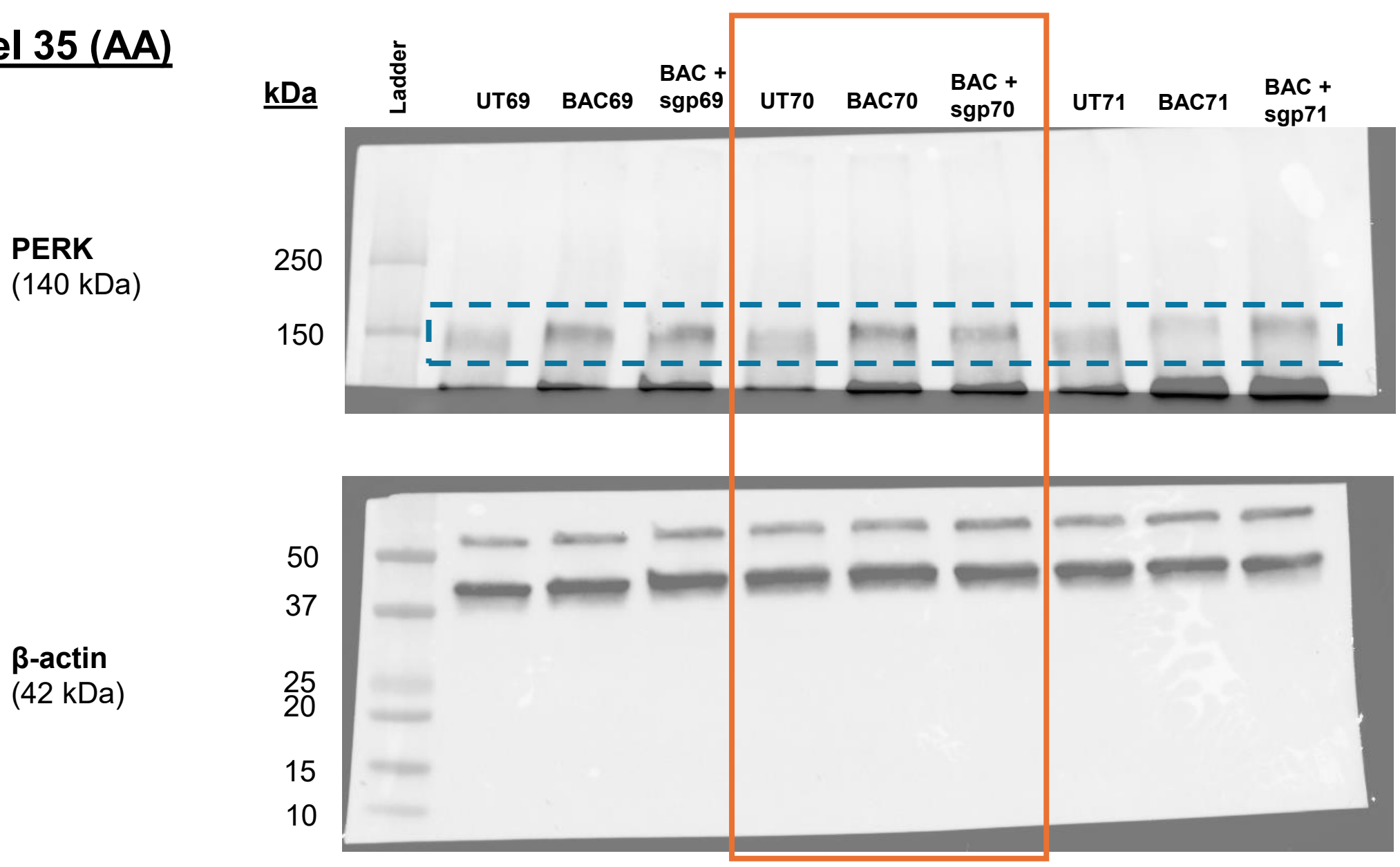

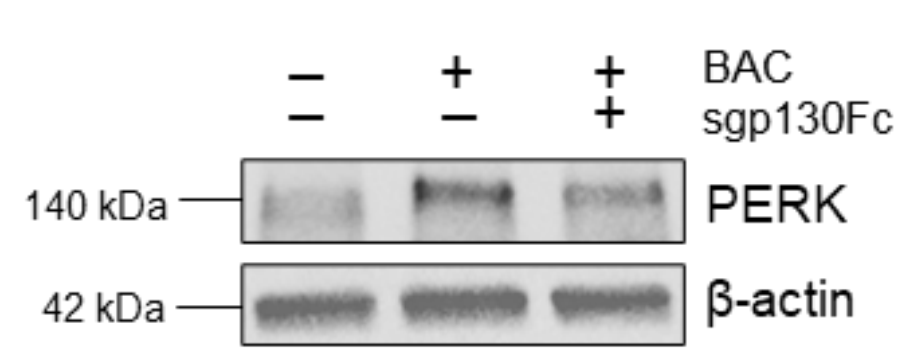

Used as representative for Figure 3D.

Raw data of Figure 3e (Ero1α)

Gel 19 (KL)

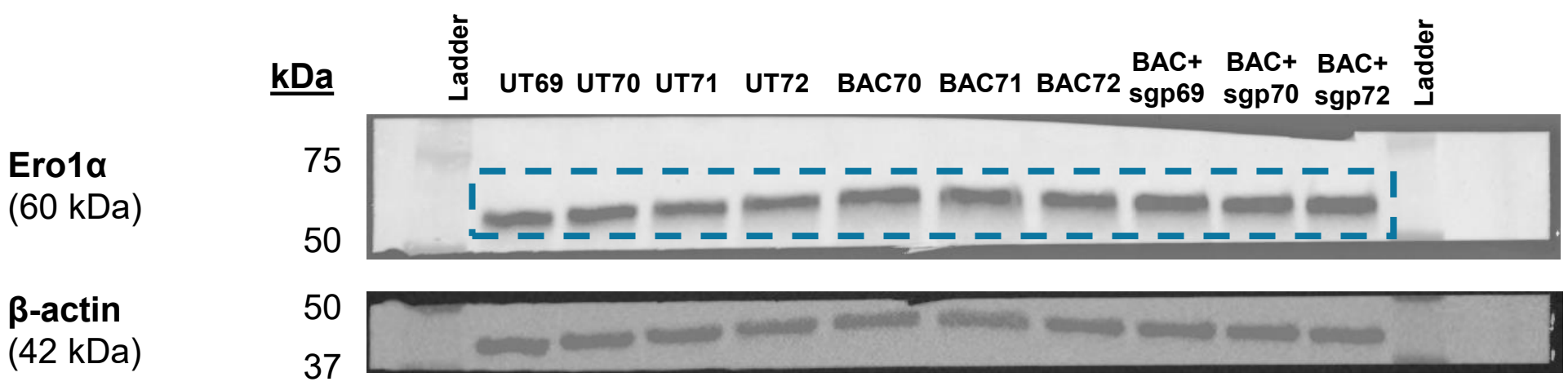

Gel 23 (KL)

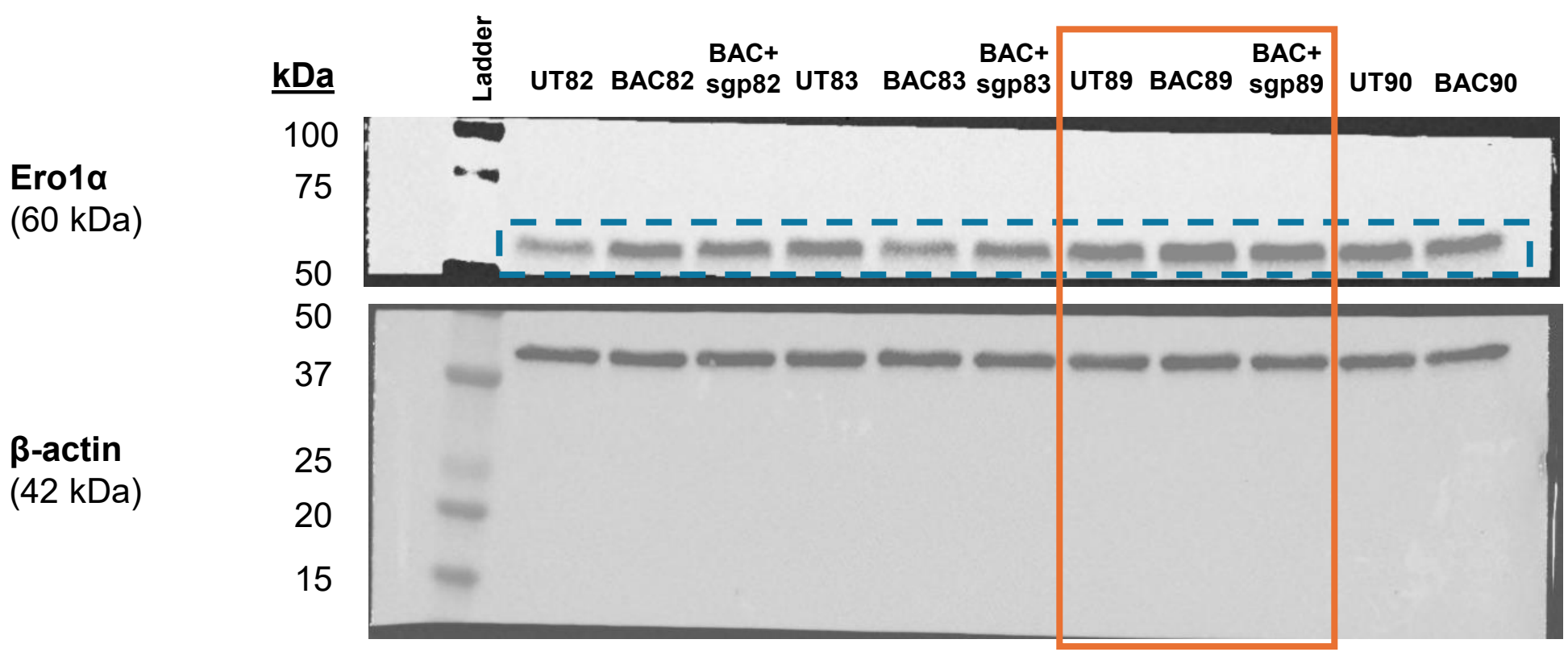

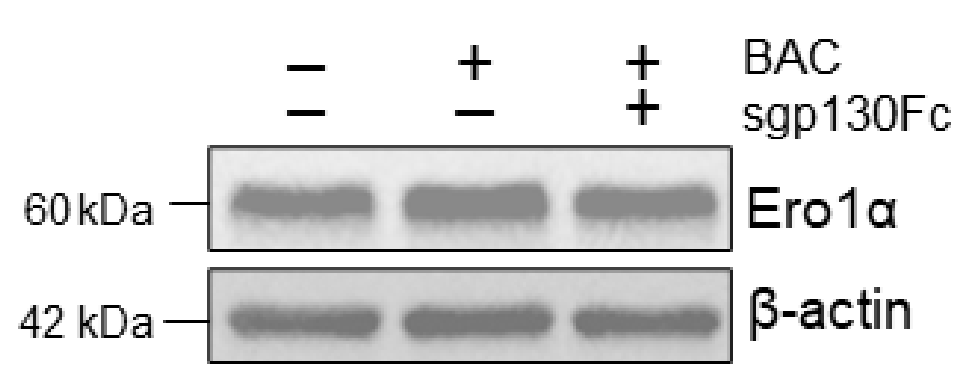

Used as representative for Figure 3E.

Raw data of Figure 4b (β-catenin, Occludin, Claudin-1)

Gel 27 (AA)

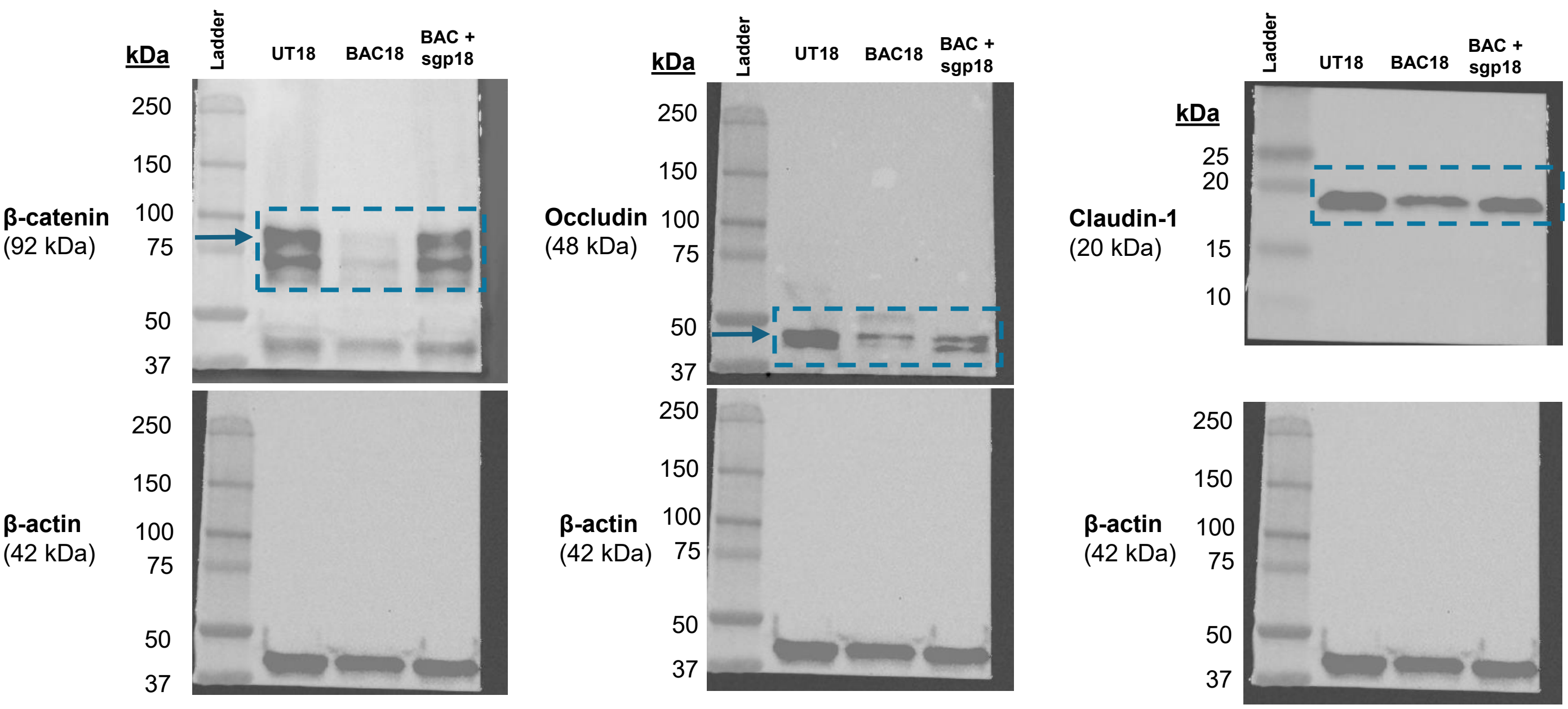

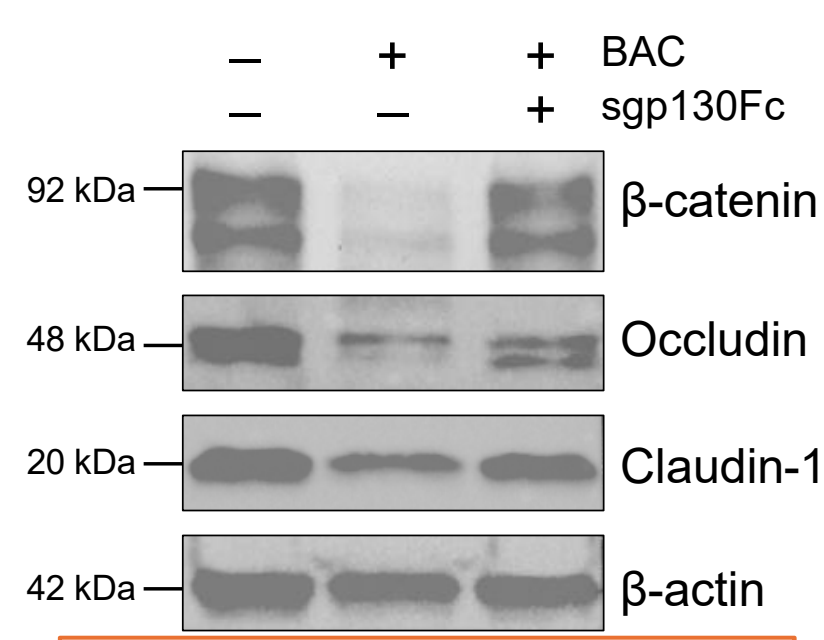

Used as representative for Figure 4B.

Raw data of Figure 4c (β-catenin)

Gel 163 (JG)

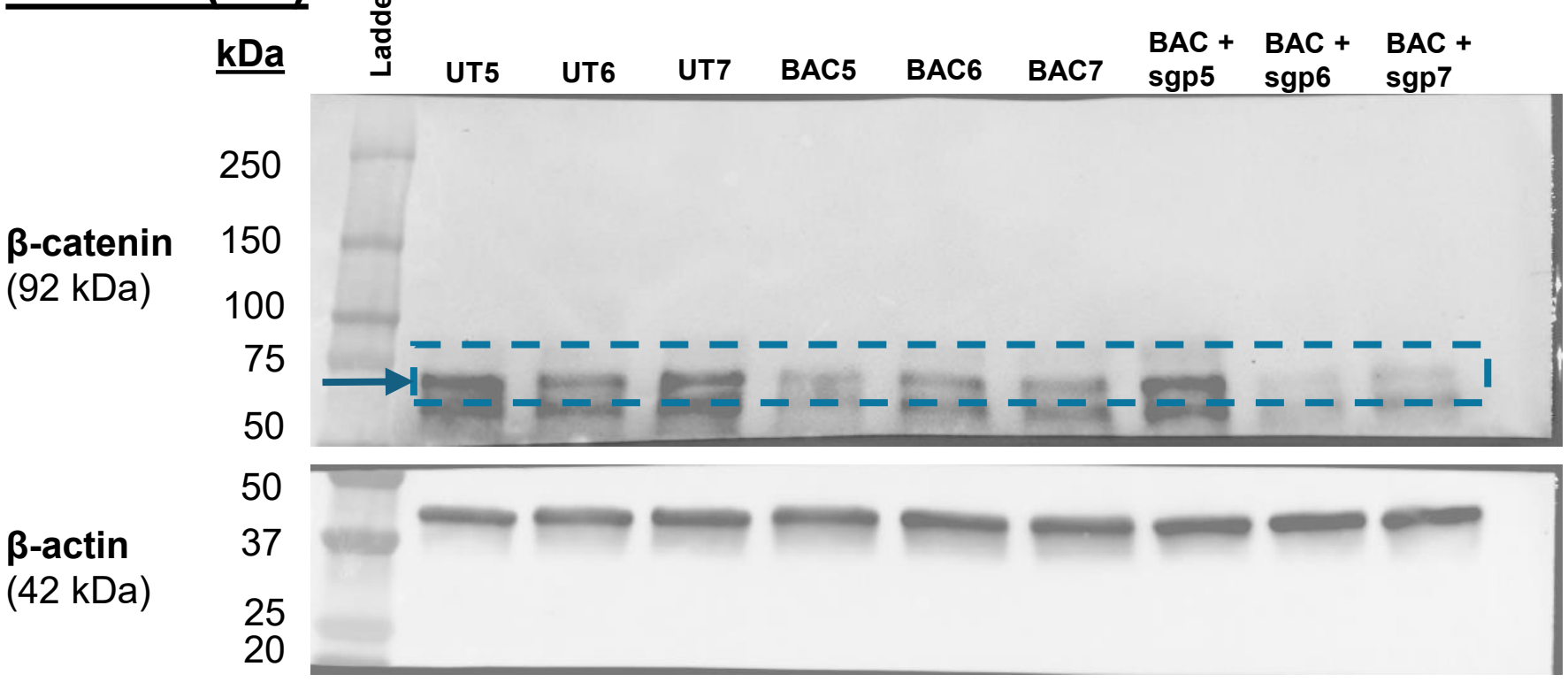

Gel 165 (JG)

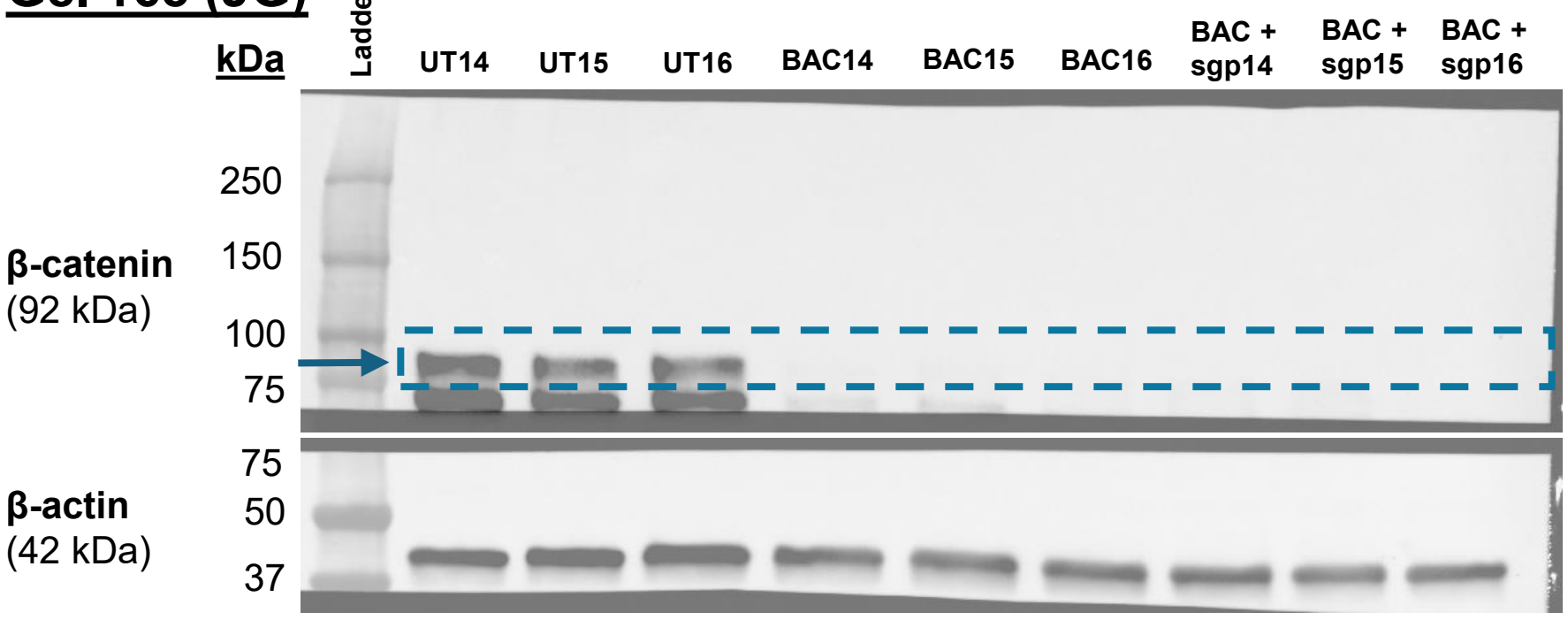

Gel 168 (JG)

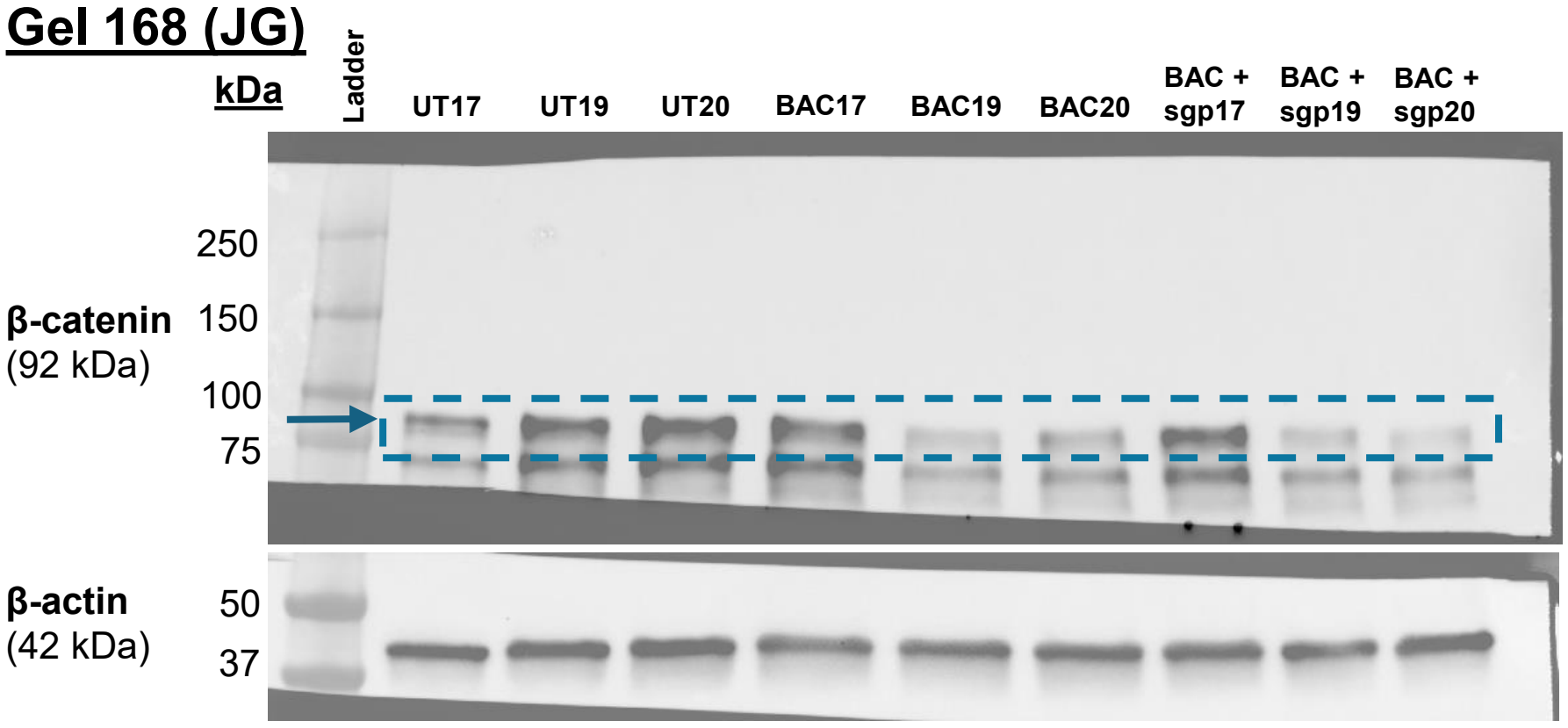

Gel 169 (JG)

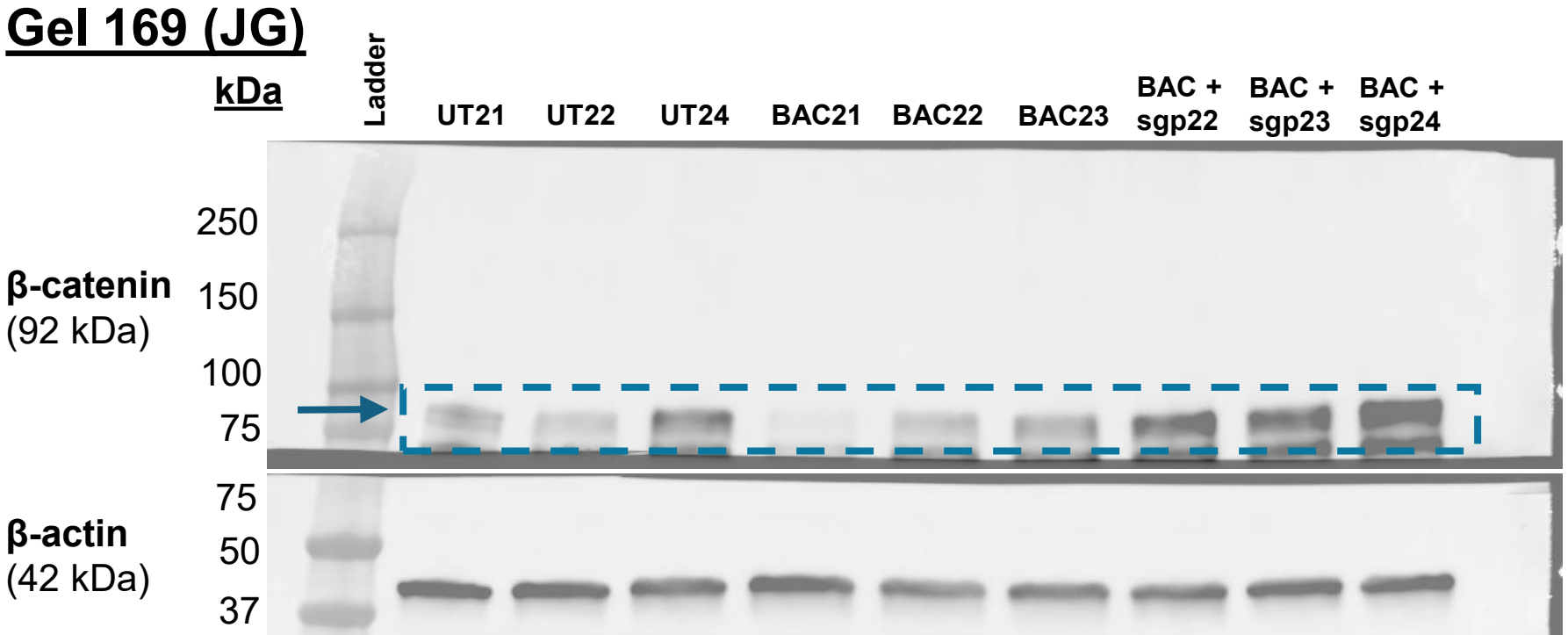

Raw data of Figure 4c (β-catenin) Continued

Gel 171 (JG)

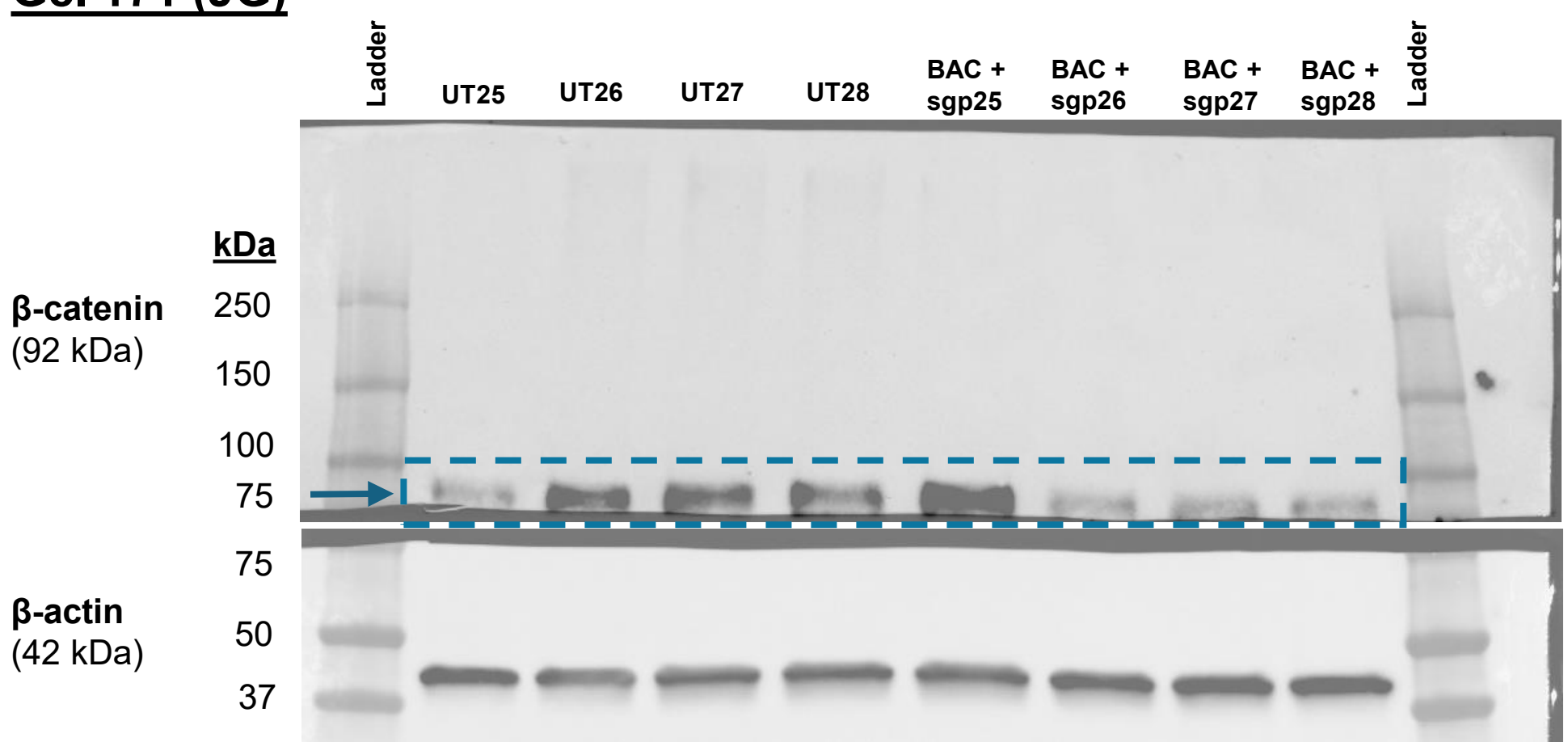

Gel 23 (AA)

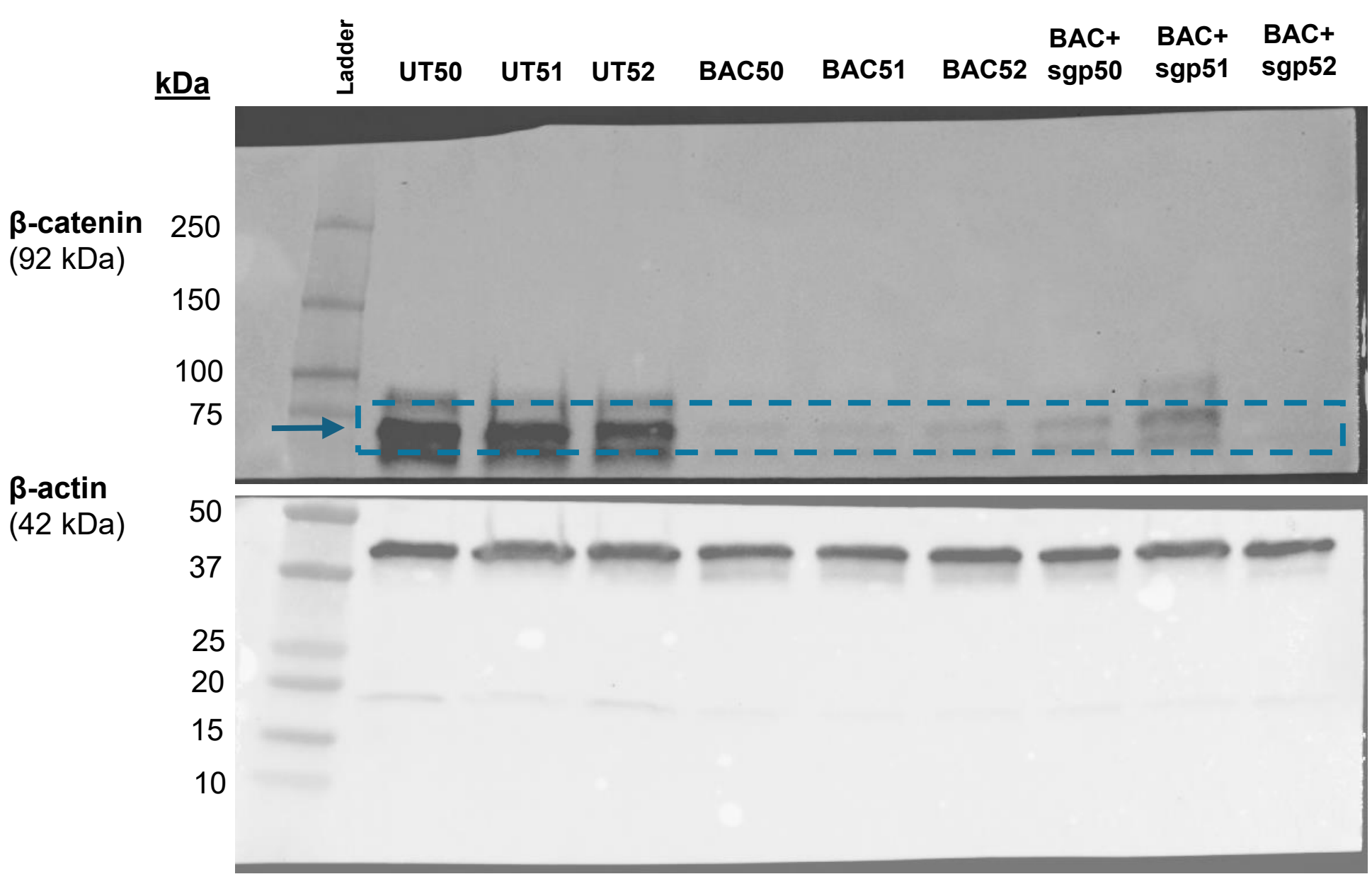

Raw data of Figure 4d (Occludin)

Gel 22 (AA)

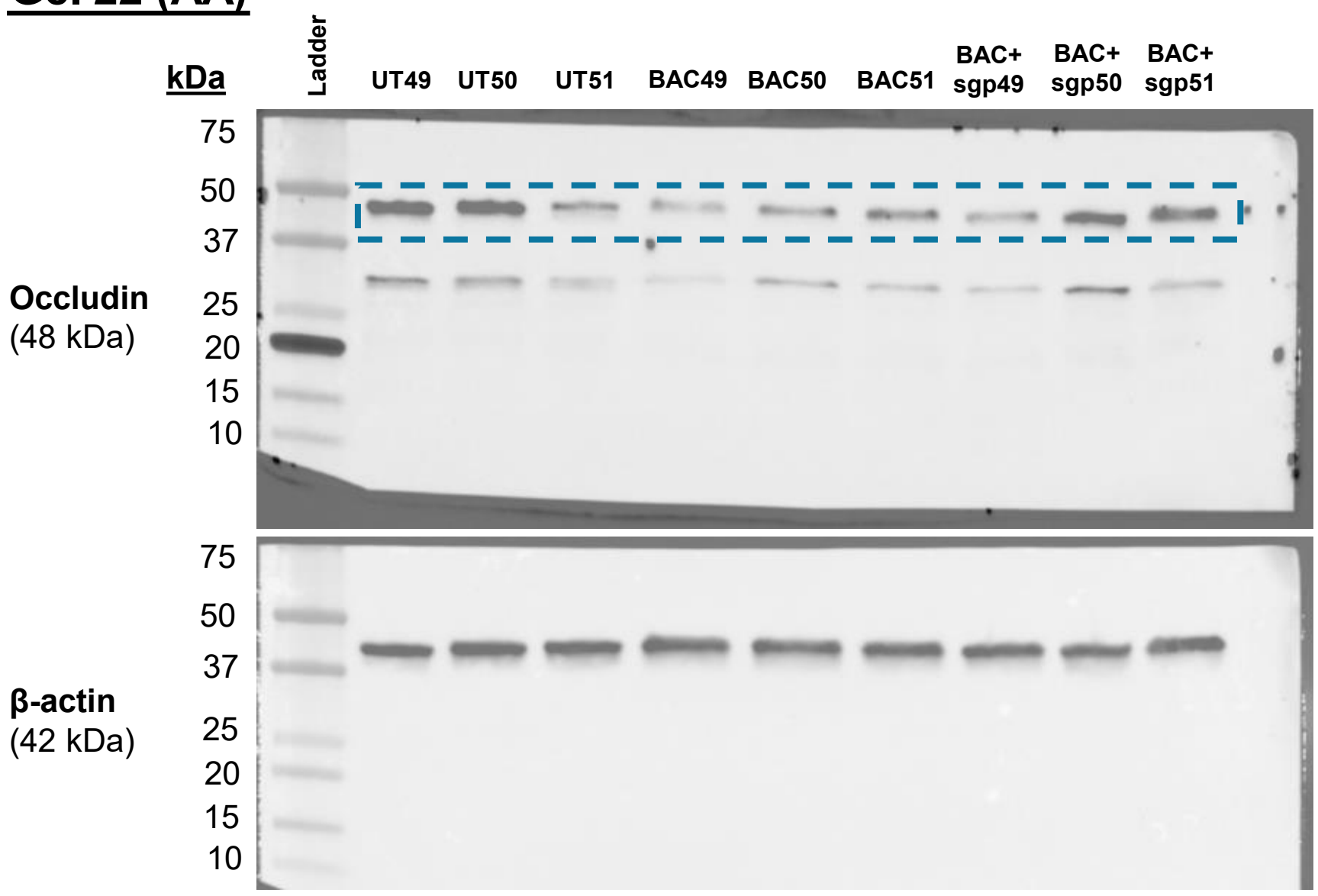

Gel 24 (AA)

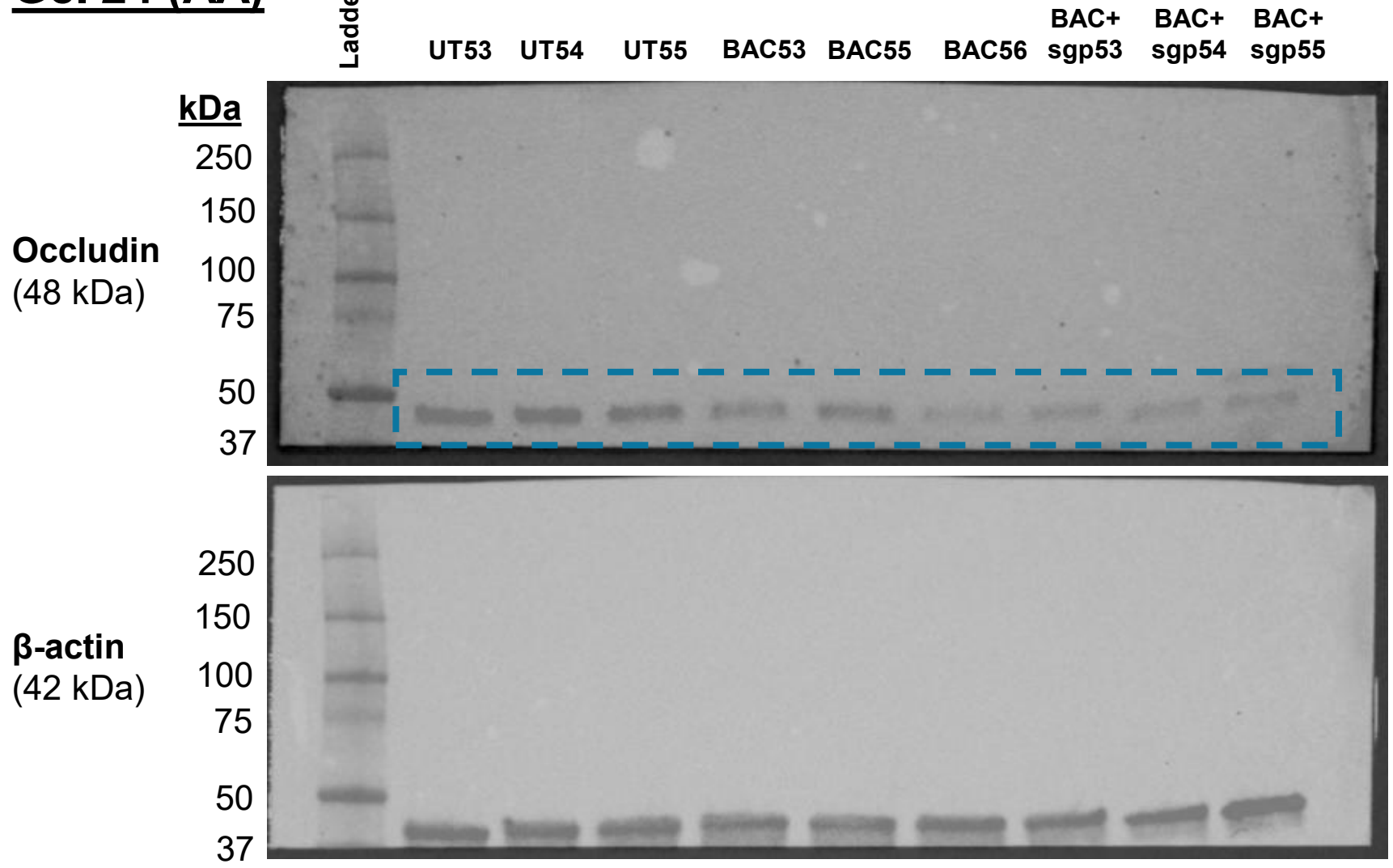

Gel 168 (JG)

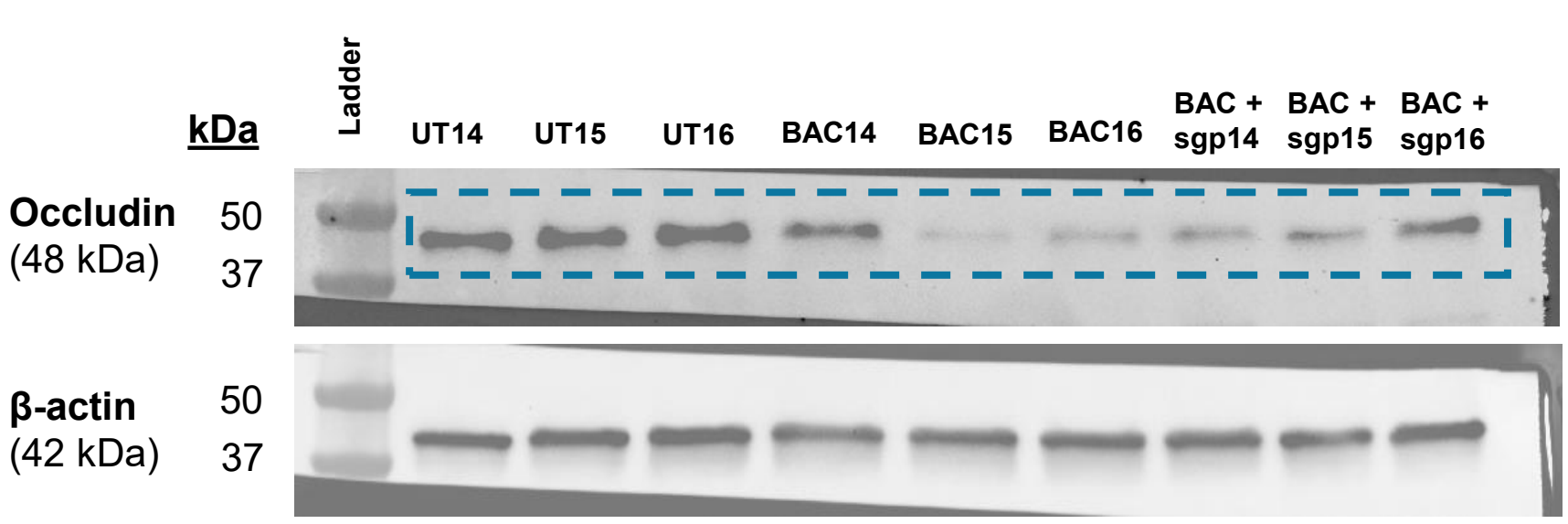

Raw data of Figure 4e (Claudin-1)

Gel 23 (AA)

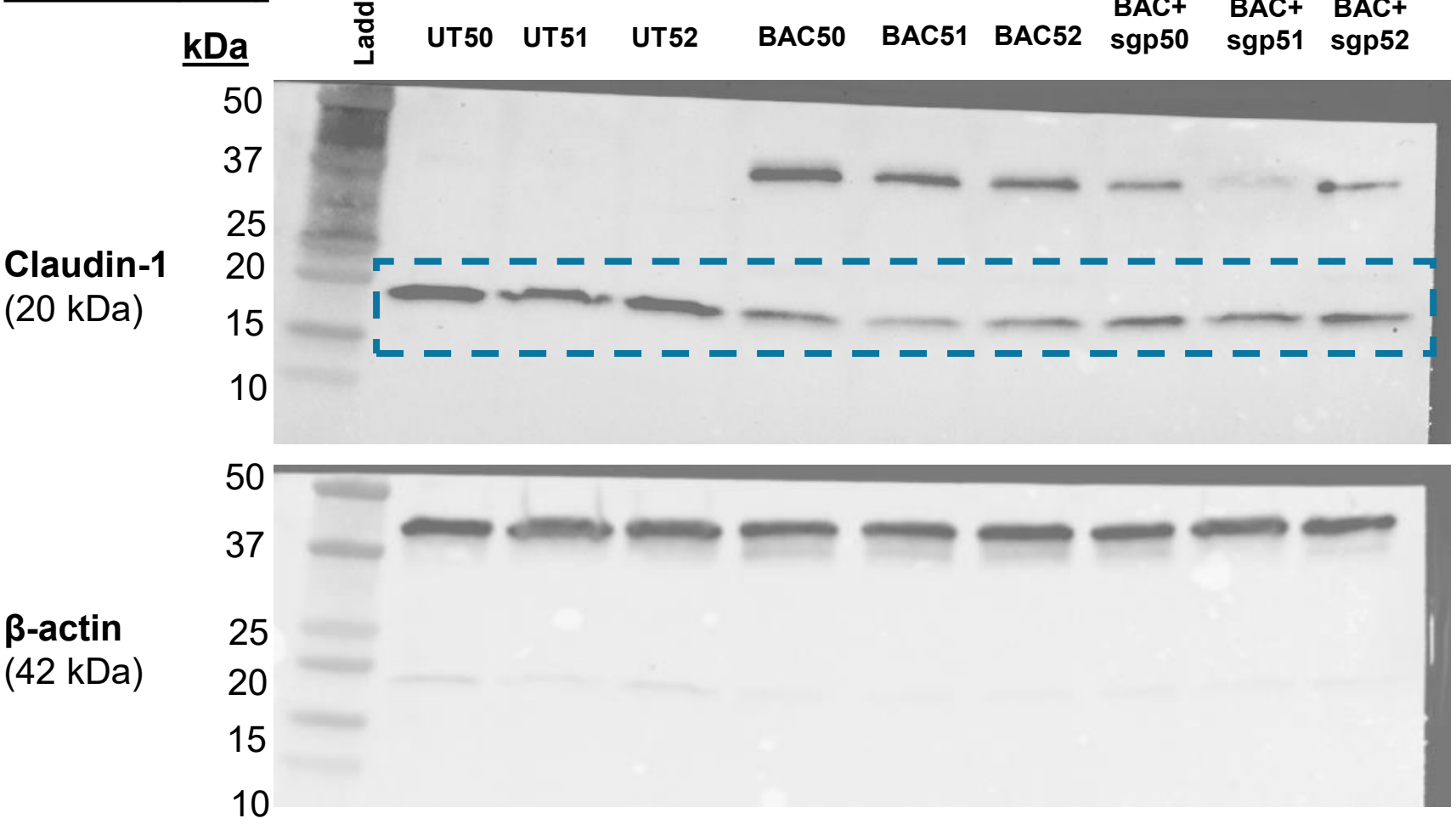

Gel 24 (AA)

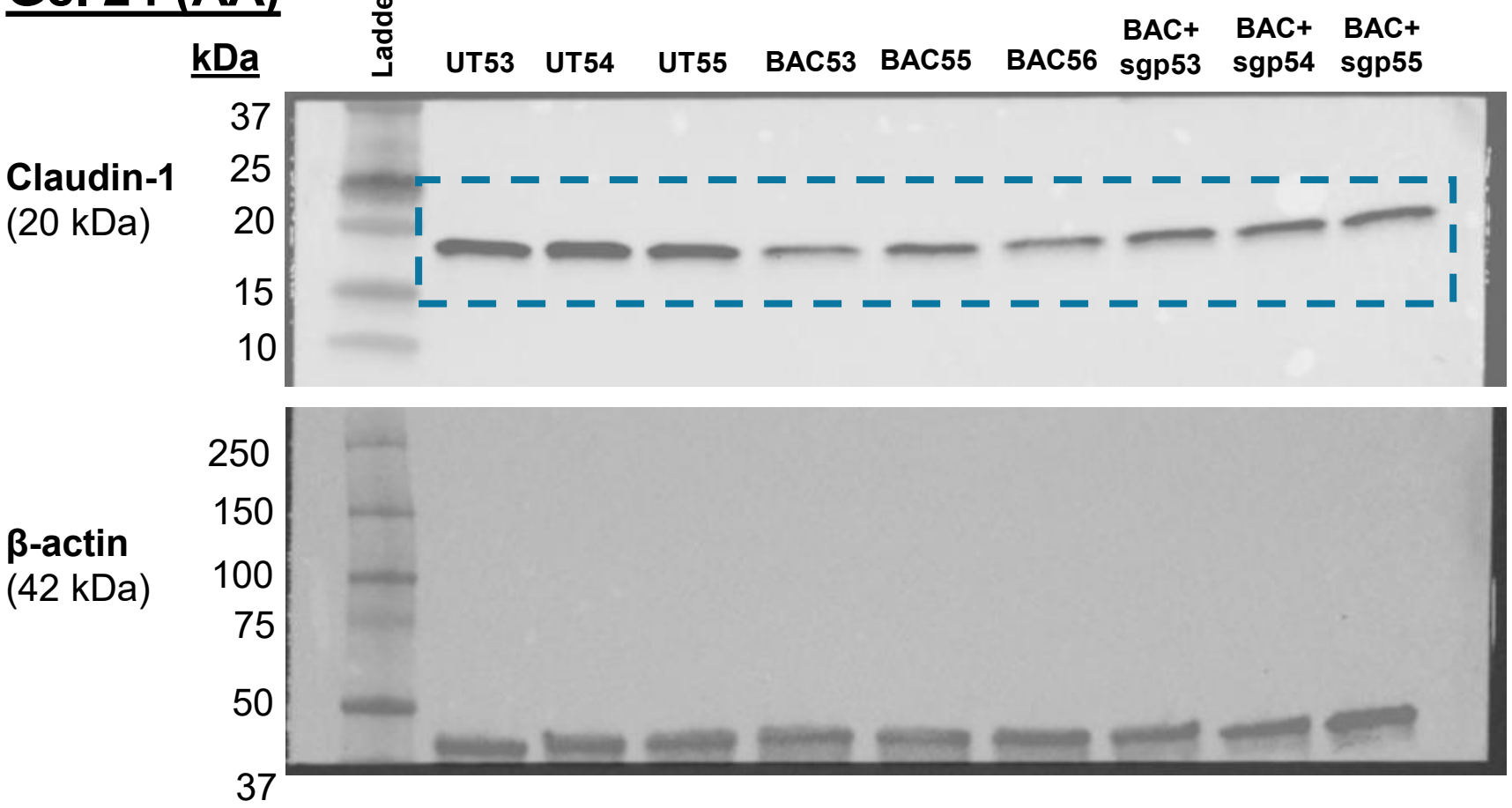

Gel 164 (JG)

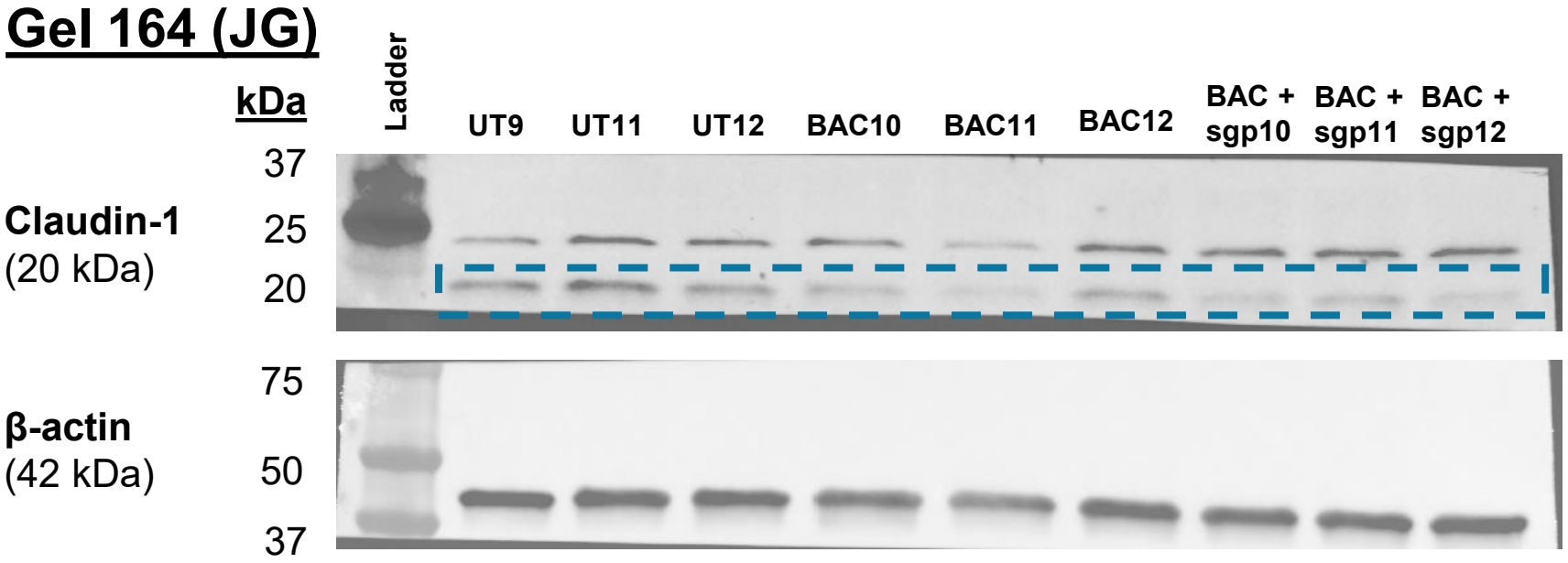

Gel 168 (JG)

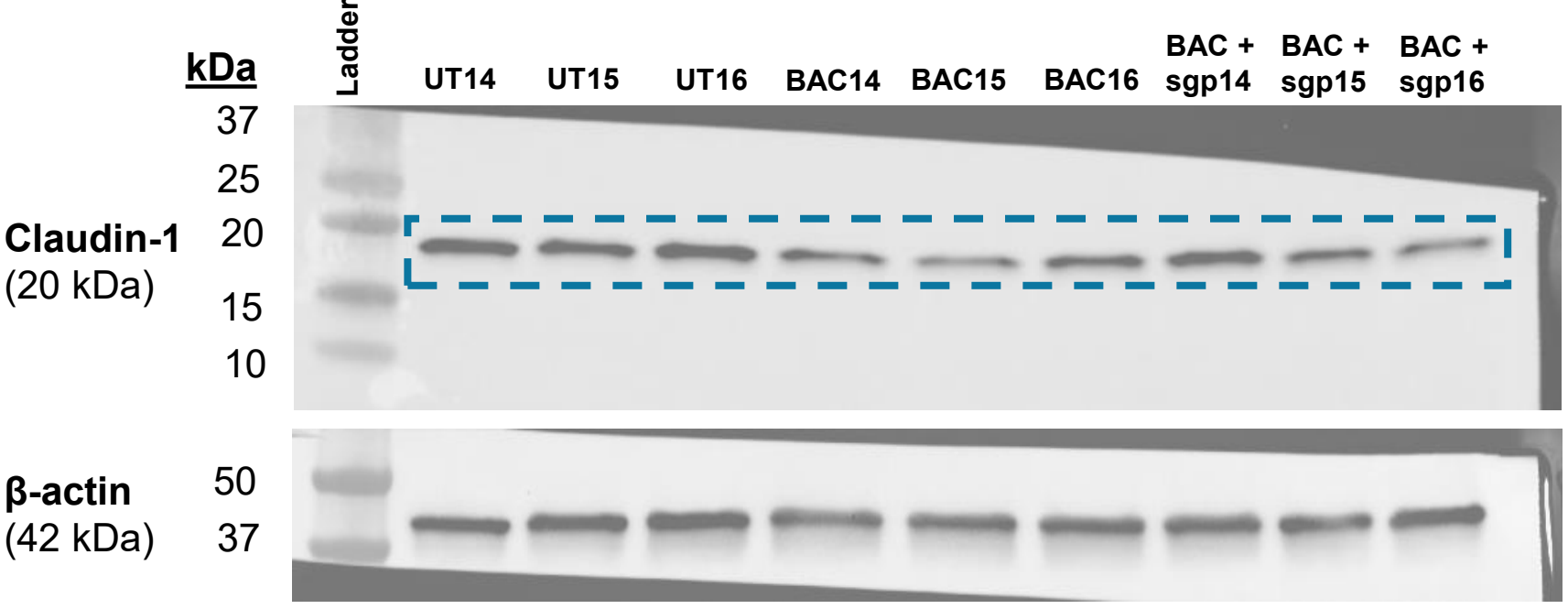

Supplement: Supplementary file 1 [file 40662_2026_513_MOESM3_ESM.pdf]
