# Supplementary material for: Blocking IL-6 trans-signaling mitigates benzalkonium chloride-induced inflammation, oxidative imbalance, ER stress, and barrier disruption in human corneal epithelial cells
Source: Eye Vis (Lond). 2026 Oct 20;13:46. doi: 10.1186/s40662-026-00513-0 (PMC13681774; doi:10.1186/s40662-026-00513-0)
Supplement: Supplementary file 2 [file 40662_2026_513_MOESM1_ESM.docx]

**Additional file 1.** Endogenous levels of membrane-bound interleukin-6 receptor (mIL-6R) and glycoprotein 130 (gp130) in untreated and benzalkonium chloride (BAC)-treated human telomerase-immortalized corneal epithelial (hTCEpi) cells. hTCEpi cells were exposed to 0.001% BAC for 30 min, and intracellular levels of IL-6 signaling receptors were assessed by western blotting. (**a, b, c**) Protein levels of mIL-6R decreased upon BAC treatment, but there was no significant change in gp130 levels. β-actin is used as loading control. Data represented as mean ± standard error of mean, n = 6/group; ***P* < 0.01, ns, not significant.
