# Supplementary material for: Blocking IL-6 trans-signaling mitigates benzalkonium chloride-induced inflammation, oxidative imbalance, ER stress, and barrier disruption in human corneal epithelial cells
Source: Eye Vis (Lond). 2026 Oct 20;13:46. doi: 10.1186/s40662-026-00513-0 (PMC13681774; doi:10.1186/s40662-026-00513-0)
Supplement: Supplementary file 3 [file 40662_2026_513_MOESM2_ESM.docx]

**Additional file 2.** Cell viability of human telomerase-immortalized corneal epithelial (hTCEpi) cells after 30-min exposure to different doses of benzalkonium chloride (BAC). Cell viability upon treatment with different doses of BAC (0.00001%, 0.0001%, 0.001%, 0.01%, 0.1%, and 1%) was determined by 3-(4,5-dimethylthiazol-2-yl)-2,5-diphenyltetrazolium bromide (MTT) assay, demonstrating ~75% cell viability for cells exposed to 0.001% BAC. Absorbance values at 540 nm were normalized to untreated (UT) hTCEpi cells for each run. Data represented as mean ± standard error of mean, n = 4/group.
